# Upcycling of Polyethylene Wastes to Valuable Chemicals over Group VIII Metal‐decorated WO_3_ Nanosheets

**DOI:** 10.1002/advs.202410574

**Published:** 2024-12-06

**Authors:** Qimin Zhou, Weiqiang Gao, Deliang Wang, Yinlong Chang, Hanxi Guan, Khak Ho Lim, Xuan Yang, Pingwei Liu, Wen‐Jun Wang, Bo‐Geng Li, Qingyue Wang

**Affiliations:** ^1^ College of Chemical and Biological Engineering Zhejiang University 866 Yuhangtang Rd Hangzhou Zhejiang 310058 P. R. China; ^2^ Institute of Zhejiang University‐Quzhou 99 Zheda Rd Quzhou Zhejiang 324000 P. R. China; ^3^ State Key Laboratory of Chemical Engineering at Zhejiang University 866 Yuhangtang Rd Hangzhou Zhejiang 310058 P. R. China

**Keywords:** dehydrogenative aromatization, group VIII metal catalysts, in‐situ spectroscopic investigation, polyethylene upcycling, WO_3_ nanosheets

## Abstract

Catalytic cracking of polyolefin wastes into valuable chemicals at mild conditions using non‐noble metal catalysts is highly attractive yet challenging. Herein it is reported that 2D tungsten trioxide (2D WO_3_) nanosheets, after decorating with group VIII metal promoters (i.e., Fe, Co, or Ni), convert high‐density polyethylene (HDPE) into alkylaromatics and olefins at low temperature and ambient pressure without using any solvent or hydrogen: 2D Ni/WO_3_ with abundant Brønsted acidic sites initiates HDPE cracking at a low temperature of 240 °C; 2D Fe/WO_3_ with low energy barrier of cyclization achieves a high HDPE conversion to 84.2% liquid hydrocarbons with a selectivity of 30.9% to aromatics at 300 °C. In‐situ spectroscopic investigations and supplementary theoretical calculations illustrate that these aromatics are formed through the cyclization of alkene intermediates. These 2D catalysts also display high efficiency in the low‐temperature cracking of single‐use commercial polyethylene wastes such as packaging bags and bottles. This work has demonstrated the high potential of 2D non‐noble metal catalysts in the efficient upcycling of waste polyolefin at mild conditions.

## Graphical abstract

Group VIII metals (i.e., Fe, Co, Ni) that decorated on the tungsten trioxide nanosheets convert the high‐density polyethylene (HDPE) waste into valuable products (e.g., aromatics, olefins) at mild conditions (240–300 °C in N_2_, no solvent, ambient pressure). Mechanistic studies reveal that olefin intermediates generated by degeneration of polymer chains are subsequently converted to cyclic intermediates, and finally to aromatics.

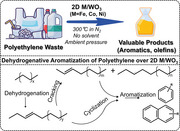

## 1 Introduction

Polyolefin wastes constitute over half of the total plastic waste, making recycling crucial for sustainable polymer industry development.^[^ ^1^, ^2^ ^]^ Catalytic cracking of polyolefins is a promising upcycling route, especially to produce aromatics that are widely applied as platform molecules to synthesize pharmaceutical drugs, surfactants, and detergents.^[^ ^3^, ^4^ ^]^ However, traditional methods involve harsh conditions to convert polyolefins to aromatics (temperatures above 500 °C or solvent use).^[^ ^5^, ^6^, ^7^ ^]^ For instance, 550 °C was required for the transition metal‐modified zeolites (i.e., HZSM‐5) to pyrolyze low‐density polyethylene (LDPE) into the mixed aromatic hydrocarbons, alkanes and olefins.^[^ ^8^ ^]^ Recent advancements include using noble‐metal catalysts to enhance polyethylene (PE) cracking efficiency at lower temperatures. For instance, 1.4 wt.% Pt/ZSM‐5 catalyst converted LDPE to monoaromatics at 450 °C under atmospheric pressure, and Pt is demonstrated to promote aromatization rates.^[^ ^9^ ^]^ Additionally, noble metal catalysts supported on metal oxides (e.g., Al_2_O_3_, ZrO_2_)^[^ ^10^, ^11^, ^12^ ^]^ exhibit high activity at lower temperatures due to synergistic effects between metal and acidic sites. Notably, 1.5 wt.% Pt/Al_2_O_3_ catalyzed PE cracking into long‐chain alkylaromatics at only 280 °C.^[^ ^10^ ^]^

Group VIII metal catalysts (e.g., Fe, Co, Ni) are commonly used for dehydrogenative aromatization of low‐molecular‐weight olefins/alkanes.^[^ ^13^, ^14^, ^15^ ^]^ Precisely engineering their metal oxide support with an optimal acid‐metal balance can intensify the dehydrogenation and cyclization of hydrocarbon chains, reducing reliance on noble catalysts. However, the cracking and aromatization of PE chains over these catalysts typically require higher temperatures (i.e., 500–650 °C).^[^ ^16^, ^17^ ^]^ Recently, we discovered that 2D WO_3_ nanosheets exhibit remarkable effectiveness in activating high‐density polyethylene (HDPE) hydrocracking—even at a low temperature of 200 °C.^[^ ^18^ ^]^ These ultrathin nanosheets, featuring abundant vacancies for anchoring metal atoms and improved catalytic site accessibility to HDPE chains, represent an ideal support platform for developing efficient catalysts for the low‐temperature cracking of polyolefin wastes. In this study, we explore 2D M/WO_3_ (M = Fe, Co, or Ni) nanosheets, possessing a monolayer ratio over 92% and atomically dispersed metal species on nanosheets. Without solvents or hydrogen, these group VIII metal‐decorated WO_3_ nanosheets catalyze HDPE cracking to aromatics and olefins at 240–300 °C. Notably, 2D Ni/WO_3_ initiates HDPE cracking at 240 °C, while Fe/WO_3_ achieves high HDPE conversion at 300 °C, yielding 84.2% liquid hydrocarbons with 30.9% selectivity to aromatics. Mechanistic insights via in‐situ DRIFTs‐Raman and theoretical calculations elucidate the HDPE‐to‐aromatics pathway, which could provide valuable guidance for the PE upcycling by non‐noble metal catalysts.

## 2 Results and Discussion

**Figure 1:**
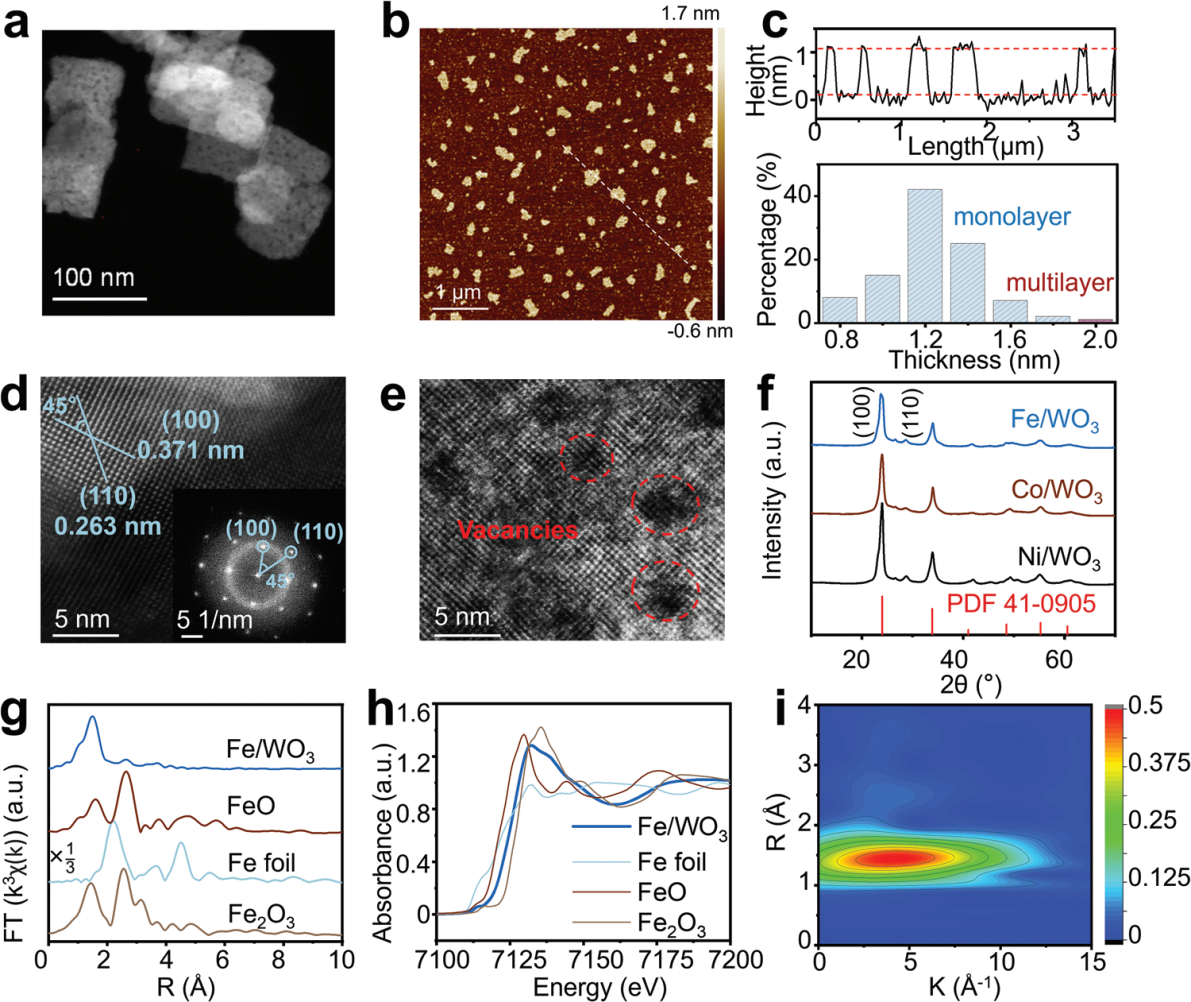
a) High‐angle annular dark‐field scanning transmission electron microscopy (HAADF‐STEM) image, b) atomic force microscope (AFM) image, c) thickness distribution, d,e) magnified HAADF‐STEM images (inset, fast Fourier transform (FFT) patterns) of WO_3_, f) X‐ray diffraction patterns of Fe/WO_3_, Co/WO_3_ and Ni/WO_3_ catalysts, g) *k* ^3^‐weighted Fourier transform spectra from extended X‐ray absorption fine structure (EXAFS) spectra of Fe/WO_3_, h) normalized X‐ray absorption near‐edge structure (XANES) spectra at the Fe *K* edge of Fe/WO_3_, Fe foil, FeO and Fe_2_O_3_, and i) wavelet‐transform (WT)‐EXAFS plots of Fe/WO_3_.

The as‐prepared WO_3_ nanosheets^[^ ^18^, ^19^ ^]^ exhibit an average lateral size of 100 ± 15 nm and an average thickness of around 1.0 nm, and the statistical data show that the monolayer ratio is over 92% (**Figure** **1**a–c). The cubic WO_3_ (001) planes are identified to be exposed on the surface of the WO_3_ nanosheets, as indicated by the characteristic d‐spacing and fast Fourier transform (FFT) patterns of the perpendicular (100) and (110) planes with a vector angle of 45° (Figure 1d).^[^ ^20^, ^21^ ^]^ Notably, abundant vacancies are observed in the high‐angle annular dark‐field scanning transmission electron microscopy (HAADF‐STEM) image (Figure 1e), facilitating the immobilization of metal species.^[^ ^18^, ^19^ ^]^ The WO_3_ nanosheets are next impregnated with saturated metal nitrate solution to prepare the 0.8 wt.% M/WO_3_ catalysts (M = Fe, Co, Ni, Table S1, Supporting Information). The obtained M/WO_3_ catalysts display similar diffraction patterns (Figure 1f) that well match with the standard patterns of cubic WO_3_ (JCPDS No. 41–0905), implying that the loaded metal species are amorphous instead of the agglomerated state.^[^ ^22^ ^]^

Extended X‐ray absorption fine structure (EXAFS) spectra are performed to verify the local geometry around the group VIII metals. Taking Fe/WO_3_ as an example, its Fourier transforms spectra from EXAFS (Figure 1g) display one prominent peak at 1.7 Å corresponding to the Fe–O with an average coordination number of 6.0; a weak peak at 2.2 Å corresponding to either the Fe–Fe or the Fe–W.^[^ ^23^, ^24^, ^25^ ^]^ These are significantly different from those of metallic iron and iron oxides, revealing that Fe is in an isolated status with the coordination of six oxygen atoms.^[^ ^26^ ^]^ On the normalized X‐ray absorption near‐edge structure (XANES) spectra (Figure 1h), the Fe *K*‐edge position of Fe/WO_3_ is located in between those of FeO and Fe_2_O_3_, suggesting that the oxidation status of Fe is in the range of +2 and +3, in agreement with its XPS spectrum (Figure S3, Supporting Information). To investigate the dispersion of Fe species, wavelet‐transform (WT) analysis is conducted by discerning the high‐resolution Fe *K*‐edge EXAFS oscillations in both *k* and *R* space. The WT contour plot of Fe/WO_3_ (Figure 1i) exhibits one maximum peak at ≈4.7 Å^−1^, distinct from Fe foil, FeO, and Fe_2_O_3_. These results confirm that Fe species are atomically dispersed on 2D Fe/WO_3_. The same conclusions also come to Co/WO_3_ (Figure S4, Supporting Information) and Ni/WO_3_ (Figure S5, Supporting Information).

**Figure 2:**
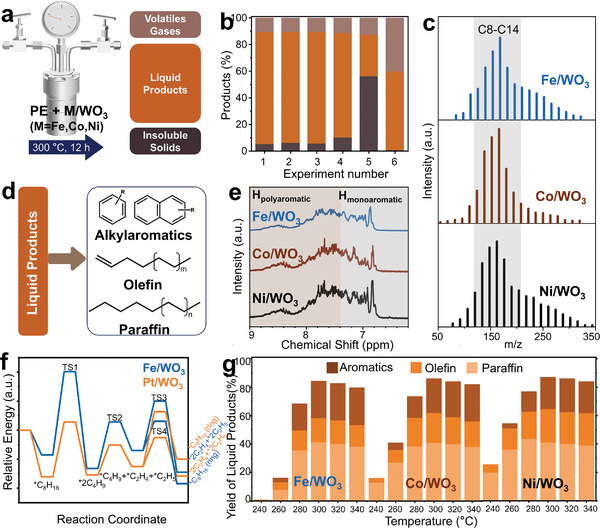
a) Schematic of reactor and product fraction for HDPE (*M_w_* = 6.6 × 10^4^ g mol^−1^, *Ð* = 4.0) cracking over M/WO_3_ nanosheets (M = Fe, Co, Ni), b) product distributions at 300 °C for 12 h catalyzed by: ① Fe/WO_3_, ② Co/WO_3_, ③ Ni/WO_3_, ④ WO_3_, ⑤ bulk Fe/WO_3_, and ⑥Pt/WO_3_, c) matrix‐assisted laser desorption/ionization coupled to time‐of‐flight mass spectra (MALDI‐TOF MS) of liquid products over M/WO_3_ nanosheets, d) schematic of liquid product constituent, e) ^1^H nuclear magnetic resonance (NMR) spectra of liquid products, f) theoretical calculation of reaction energy for octane cracking over Fe/WO_3_ and Pt/WO_3_, g) catalytic performance of M/WO_3_ nanosheets in HDPE cracking as a function of reaction temperatures from 240 °C to 340 °C.

Since HDPE has a denser structure than LDPE, it is more difficult to decompose under the same conditions.^[^ ^10^, ^27^ ^]^ We chose HDPE with a high molecular weight (*M_w_* = 6.6 × 10^4^ g mol^−1^, *Ð* = 4.0) as the sample to demonstrate the effectiveness of 2D M/WO_3_ catalysts. Catalytic cracking of the HDPE is performed in a stirred mini‐autoclave under ambient pressure in N_2_ without solvent (**Figure** **2**a). Specifically, HDPE is converted primarily to liquid hydrocarbons (≈85%) over M/WO_3_ nanosheets (M = Fe, Co, or Ni) at 300 °C for 12 h with approximately 10% volatile products (Experiment 1–3, Figure 2b). The matrix‐assisted laser desorption/ionization coupled to time‐of‐flight mass spectrometry (MALDI‐TOF MS) results disclose that the cracking liquid products of HDPE over M/WO_3_ nanosheets are predominantly C8‐C14 hydrocarbons (Figure 2c). They consist of aromatics, olefins, and alkanes, as determined by gas chromatography (Figure 2d; Figures S6–S8, Supporting Information). The ^1^H nuclear magnetic resonance (NMR) spectra of liquid products (Figure 2e) suggest the presence of substantial aromatic hydrocarbons (H_ar_) (6.5 to 9.0 ppm), including monoaromatic hydrocarbons (6.5 to 7.4 ppm) and polyaromatic hydrocarbons (7.4 to 9.0 ppm).^[^ ^10^ ^]^ Protons associated with an aliphatic carbon directly bonded to an aromatic ring (H_α_) show up in the region 2 to 4 ppm, and the H_α_/H_ar_ ratio is 2.32, suggesting that the aromatic species mainly contain tri‐alkylaromatics and tetra‐alkylnaphthalenes (Figures S9 and S10, Supporting Information).

For comparison, 2D WO_3_ nanosheets (Experiment 4), Fe decorated bulk WO_3_ −(3D Fe/WO_3_, Experiment 5), and Pt/WO_3_^[^ ^18^ ^]^ (Experiment 6) are also studied as reference catalysts in the catalytic cracking of HDPE under the same conditions (Figure 2b). Compared to 2D M/WO_3_ nanosheets, a lower HDPE conversion of 90.0% with less aromatics is obtained on 2D WO_3_ nanosheets (Experiment 4, Table S4, Supporting Information). This observation implies that the WO_3_ nanosheets with well‐distributed group VIII metal species promote the dehydrogenative aromatization of HDPE. A HDPE conversion of 44.0% with a yield of aromatics at 1.9% is attained on 3D bulk Fe/WO_3_ (Experiment 5), which is far inferior to that of 2D Fe/WO_3_ (94.9%). It has been reported ^[^ ^18^ ^]^ that defective 2D monolayer WO_3_ with large surface area (Figure S11 and Table S5, Supporting Information) allows metal sites to anchor atomically (Figure 1), and further enhances the activation of polymer chains (via adsorption) and surface transfer of the active intermediates. Almost all the HDPE is decomposed by 2D Pt/WO_3_, but with only 59.2% of liquid hydrocarbons and 40.3% of volatile light hydrocarbons, for which the major products are low‐value C3−C36 alkanes (76.2%) and almost no aromatics (<0.1%) (Experiment 6, Figure S12, Supporting Information). These results could be understood from the calculation by density functional theory (Figure 2f; Figure S13, Supporting Information, octane is used as a simplified model of PE^[^ ^28^, ^29^ ^]^): Fe/WO_3_ prefers to go through cyclization to generate C_8_H_16_ ring with a lower barrier of 0.40 eV instead of secondary cracking of 0.68 eV, thus promoting the aromatization; while Pt/WO_3_ follows the deep cracking with a lower barrier of 0.60 eV than cyclization of 0.75 eV.

The catalytic characteristics (i.e., low‐temperature activation, aromatization) of Fe/WO_3_, Co/WO_3_, and Ni/WO_3_ are further investigated individually under different HDPE cracking temperatures ranging from 240 °C to 340 °C (Figure 2g). Remarkably, Ni/WO_3_ initiated HDPE cracking at a low temperature of 240 °C with a conversion of 25.6%, followed by Co/WO_3_ of 16.2%, while Fe/WO_3_ barely converted any HDPE at 240 °C (Figure 2g). The conversion steadily increases along with the rising temperatures, with enhancing yields of aromatic products. Ultimately, all these catalysts converted HDPE to yield of around 85% liquid hydrocarbons at 300 °C, of which the highest selectivity to aromatics at 30.9% of Fe/WO_3_, followed by 27.9% of Co/WO_3_ and 25.6% of Ni/WO_3_. The Fe/WO_3_ nanosheets also exhibit a good reusability with less than 3.1% conversion drop after 5 cycles (Figure S14, Supporting Information).

**Figure 3:**
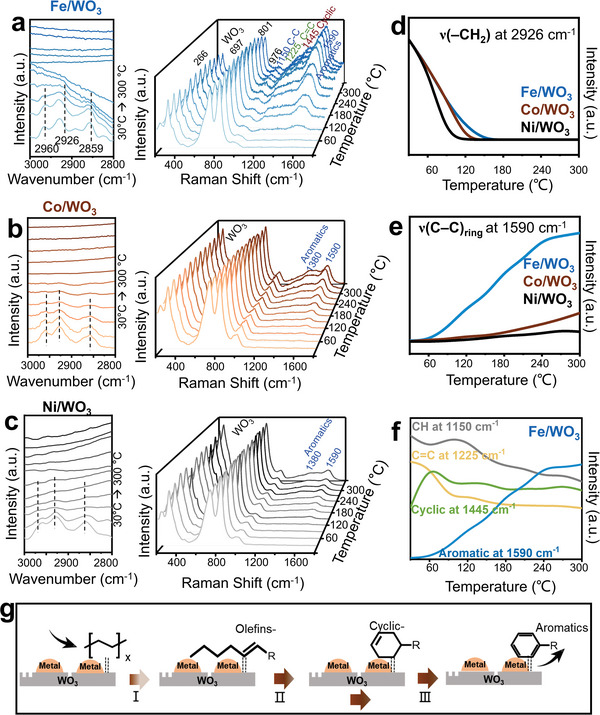
In‐situ diffuse reflectance infrared transform spectroscopy (DRIFT) spectra (left) and in‐situ micro‐Raman spectra (right) of octane catalytic cracking over a) Fe/WO_3_, b) CoWO_3_, and c) Ni/WO_3_; dynamic intensity changes of d) ν(−CH_2_) at 2926 cm^−1^ and e) ν(C‐C)_ring_ at 1590 cm^−1^ over M/WO_3_ (M = Fe, Co, Ni), f) dynamic changes of characteristic vibrations of Fe/WO_3_ as a function of reaction temperatures, and g) proposed reaction pathways of catalytic cracking over Fe/WO_3_ (I: cracking; II: cyclization; III: aromatization).

In‐situ diffuse reflectance infrared transform spectroscopy (DRIFT) and in‐situ micro‐Raman investigations are performed over Fe/WO_3_, Co/WO_3,_ and Ni/WO_3_ for the catalytic cracking of the simplified model of HDPE (i.e., octane^[^ ^28^, ^29^ ^]^) to unveil the reaction pathway. The assignments of the characteristic vibrations are listed in Tables S6 and S7 (Supporting Information). It shows the adsorbed alkane reactant on M/WO_3_ (ν_s_(CH_3_) at 2960 cm^−1^, ν_s_(CH_2_) at 2926 cm^−1^ and ν_s_(C–H) at 2859 cm^−1^, **Figure** **3**a–c ^[^ ^30^, ^31^ ^]^) is gradually consumed as the reaction temperature increases from 30 °C to 300 °C Simultaneously, various intermediates accumulate on M/WO_3_ nanosheets, including olefin (ν(C = C) at 1225 cm^−1^), cycloalkene (ν_cyclo_(C‐C) at 1445 cm^−1^) and aromatic (ν_ring_(C–C) at 1590 cm^−1^, ν_ring_(CH_3_) at 1380 cm^−1^, Figure 3a–c).^[^ ^32^, ^33^ ^]^

Figure 3d,e plots the dynamic intensity changes over the three M/WO_3_ nanosheets during the heating process of ν_s_(CH_2_) at 2926 cm^−1^ and ν_ring_(C–C) at 1590 cm^−1^, respectively. Notably, Ni/WO_3_ exhibits the highest activity at low temperatures with the disappearance of adsorbed alkane at 120 °C, followed by Fe/WO_3_ at 150 °C and Co/WO_3_ at 180 °C (Figure 3d). Meanwhile, more aromatics are formed on Fe/WO_3_, compared to those on Co/WO_3_ and Ni/WO_3_ (Figure 3e). These results demonstrate the higher low‐temperature cracking activity of Ni/WO_3_ while the better aromatization ability of Fe/WO_3_, consistent with their HDPE cracking performance. Figure 3f further plots the dynamic intensity changes of the intermediates for olefins (ν(C = C) at 1225 cm^−1^), cycloalkenes (ν(C‐C) at 1445 cm^−1^) and aromatics (ν_ring_(C–C) at 1590 cm^−1^) over Fe/WO_3_ nanosheets during the heating process. It reveals that olefin intermediates generated on Fe/WO_3_ at low temperatures, are subsequently converted to cyclic intermediates, and finally to aromatics (Figure 3g).^[^ ^34^ ^]^

**Figure 4:**
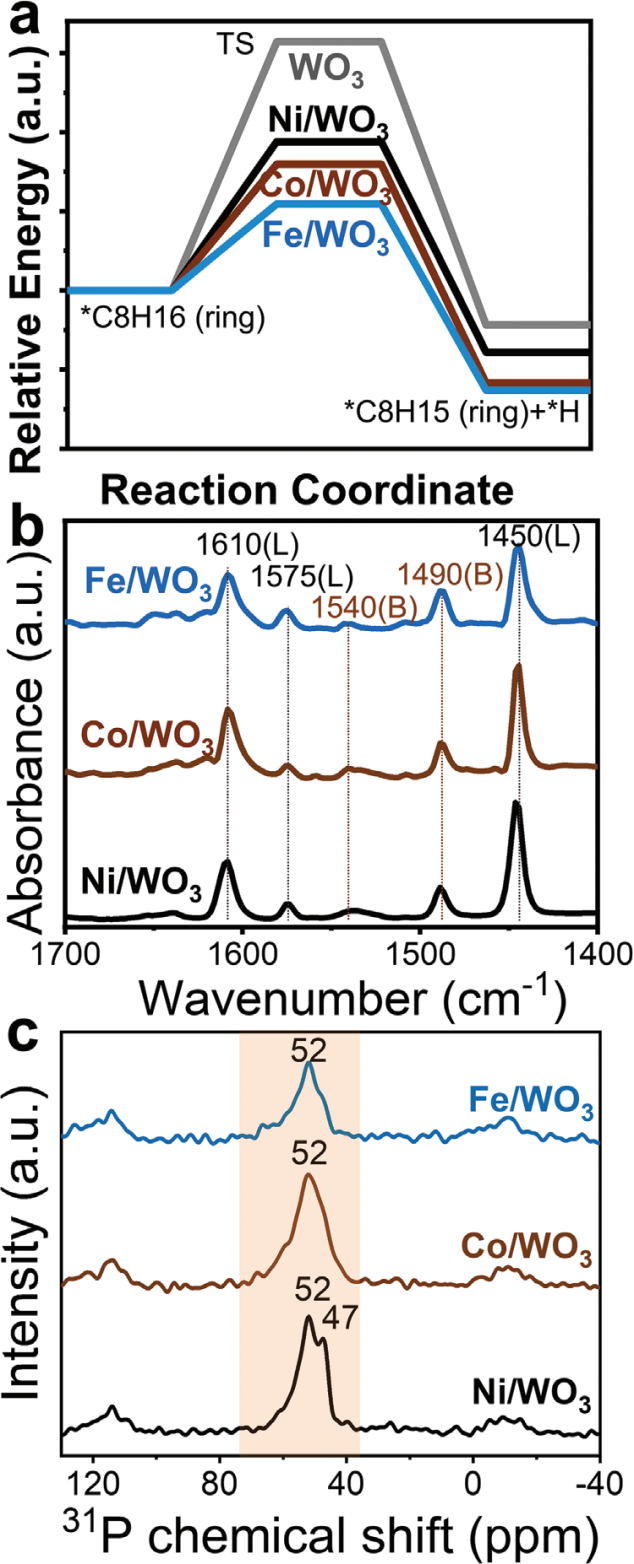
a) Reaction energy diagram of cyclooctane dehydrogenation to the alkylbenzene, b) pyridine infrared spectra, and c) ^31^P nuclear magnetic resonance (NMR) spectra of Fe/WO_3_, Co/WO_3_ and Ni/WO_3_.

Dehydrogenation is proposed to be the selectivity‐controlling step of dehydrogenative aromatization in HDPE cracking.^[^ ^35^, ^36^, ^37^ ^]^ Transition states of the dehydrogenation and their corresponding energy barrier (i.e., cyclic intermediates go through dehydrogenation to form alkyl‐benzene) are thus calculated for the three M/WO_3_ nanosheets and undecorated WO_3_ nanosheets (see Section S1.6, Supporting Information). **Figure** **4**a indicates a much lower energy barrier of 0.54 eV on Fe/WO_3_ for the dehydrogenation of C8 cyclic intermediates, as compared to 0.79 eV of Co/WO_3_, 0.94 eV of Ni/WO_3_, and 1.57 eV of WO_3_ nanosheets. This elucidates the higher aromatics yield of 2D Fe/WO_3_ in the catalytic HDPE cracking. Moreover, the high aromatization efficiency of Fe/WO_3_ is also associated with its high ratio of Lewis acidic sites to Brønsted acidic sites (L/B ratio) as 10, compared to Co/WO_3_ (cf. 7.5) and Ni/WO_3_ (cf. 6.5) (Figure 4b; Table S8, Supporting Information).^[^ ^38^ ^]^

The extraordinary low‐temperature activity of Ni/WO_3_ could be illustrated by its enhancive Brønsted acidic sites (BAS), which can provide hydrogen protons that initiate the cracking of PE chains.^[^ ^39^, ^40^ ^]^ The ^31^P NMR using trimethylphosphine oxide as adsorbed molecules is utilized to characterize the BAS of M/WO_3_ nanosheets (Figure 4c).^[^ ^41^ ^]^ Besides the BAS at 52 ppm that exhibits for all the three M/WO_3_ catalysts, an additional peak at 47 ppm is observed on Ni/WO_3_, determining its stronger BAS that facilitates the cracking of PE chains at low temperatures.^[^ ^38^, ^42^ ^]^

**Figure 5:**
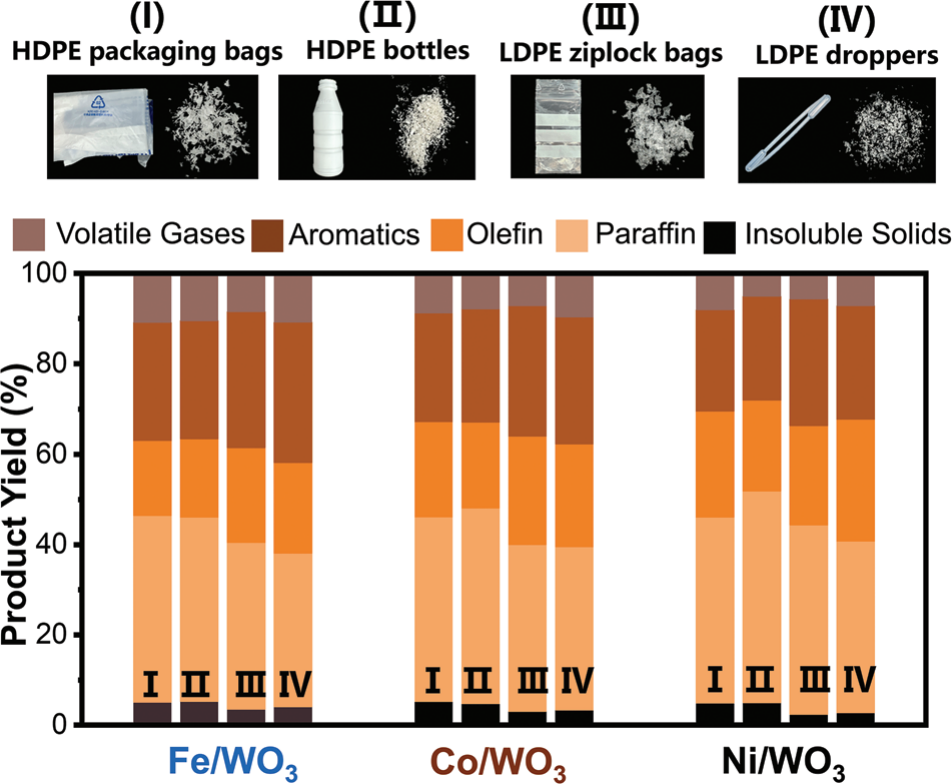
Catalytic cracking of commercial polyolefin products over Fe/WO_3_, Co/WO_3_ and Ni/WO_3_ catalysts: (I) HDPE packaging bags, (II) HDPE bottles, (III) LDPE ziplock bags, (IV) LDPE droppers.

The Fe/WO_3_, Co/WO_3_, and Ni/WO_3_ nanosheets are further examined for the cracking of various waste polyolefin products, including HDPE packaging bags, HDPE bottles, LDPE ziplock bags, and LDPE droppers (**Figure** **5**). The cracking is conducted at 300 °C under ambient pressure without solvents or hydrogen. It is interesting to note that the cracking of commercial LDPE generally displays a slightly higher conversion (96.7 ± 0.8%) than that of commercial HDPE (95.0 ± 0.3%). A high conversion to 91.9% liquid products is achieved by Ni/WO_3_ for LDPE ziplock bags. This can be attributed to the loose structure and weak chain entanglement of LDPE with characteristic branching chains, which facilitate the contact between catalyst and polymer chains.^[^ ^27^ ^]^ Moreover, Fe/WO_3_ exhibits the highest aromatization ability, yielding 31.1% selectivity to aromatics among 85.2% liquid hydrocarbons for the LDPE droppers. These results further underscore the high efficiency of Fe/WO_3_ in converting polyolefin plastics into aromatics. The catalytic cracking of commercial polyolefin plastics over low‐cost group VIII metal catalysts at mild reaction conditions, validates a novel approach for converting plastic waste into value‐added chemicals.

## 3 Conclusion

In this work, we develop a group of non‐noble M/WO_3_ catalysts (M = Fe, Co, Ni) to one‐step convert PE wastes into valuable products at mild conditions (i.e., 300 °C, no solvent, no hydrogen). Ni/WO_3_ initiates HDPE cracking at a low temperature of 240 °C, while Fe/WO_3_ produces 84.2% liquid hydrocarbons with 30.9% selectivity to aromatics at 300 °C. Comparative investigations demonstrate that isolated Fe, Co, and Ni species anchored on monolayer WO_3_ nanosheets not only enhance cracking activity, but also improve the selectivity toward aromatic products. Mechanistic studies combing in‐situ DRIFTs, in‐situ Raman studies, acidic sites analysis, and theoretical calculations disclose that Ni enhances cracking activity for its enhancive BAS, while Fe promotes PE depolymerization into olefins and their cyclization into aromatics. This strategy has been validated in converting both HDPE and LDPE commercial products, thus paving the way for developing non‐noble metal‐based cracking catalysts for efficiently upcycling waste polyolefins.

## Conflict of Interest

The authors declare no conflict of interest.

## Supporting information



Supporting Information

## Data Availability

The data that support the findings of this study are available in the supplementary material of this article.
