# Supplementary material for: Upcycling of Polyethylene Wastes to Valuable Chemicals over Group VIII Metal‐decorated WO3 Nanosheets
Source: Adv Sci (Weinh). 2024 Dec 6;12(4):2410574. doi: 10.1002/advs.202410574 (PMC11789578; doi:10.1002/advs.202410574)
Supplement: Supplementary file 1 — Supporting Information [file ADVS-12-2410574-s001.docx]

Supporting Information
©Wiley-VCH 2022
69451 Weinheim, Germany

Upcycling of Polyethylene Wastes to Valuable Chemicals over Group VIII Metal-decorated WO_3_ Nanosheets

Qimin Zhou,^#[a],[b]^ Weiqiang Gao,^#[a],[b]^ Deliang Wang,^#[a],[b]^ Yinlong Chang,^#[a],[b]^ Hanxi Guan,^[a],[b]^ Khak Ho Lim,^[a],[b]^ Xuan Yang,^[a]^ Pingwei Liu,*^[a],[c]^ Wen-Jun Wang,^[a],[c]^ Bo-Geng Li,^[a],[c]^ and Qingyue Wang*^[a],[b]^

[#] These authors contributed equally to this work.

[a] Dr. Q. Zhou, W. Gao, Prof. Dr. D. Wang, Y. Chang, Dr. H. Guan, Prof. Dr. K. H. Lim, Prof. Dr. X. Yang, Prof. Dr. P. Liu, Prof. Dr. W.-J. Wang, Prof. Dr. B.-G. Li, Prof. Dr. Q. Wang,

College of Chemical and Biological Engineering,

Zhejiang University,

866 Yuhangtang Rd, Hangzhou 310058, Zhejiang, P.R. China

E-mail: qingyuewang@zju.edu.cn; liupingwei@zju.edu.cn

[b] Dr. Q. Zhou, W. Gao, Prof. Dr. D. Wang, Y. Chang, Dr. H. Guan, Prof. Dr. K. H. Lim, Prof. Dr. Q. Wang,

Institute of Zhejiang University-Quzhou,

99 Zheda Rd, Quzhou 324000, Zhejiang, P.R. China

[c] Prof. Dr. P. Liu, Prof. Dr. W.-J. Wang, Prof. Dr. B.-G. Li,

State Key Laboratory of Chemical Engineering at Zhejiang University,

866 Yuhangtang Rd, Hangzhou 310058, Zhejiang, P.R. China

Supporting information for this article is given via a link at the end of the document.

**Abstract:** Catalytic cracking of polyolefin wastes into valuable chemicals at mild conditions using non-noble metal catalysts is highly attractive yet challenging. Herein we report that two-dimensional tungsten trioxide (2D WO_3_) nanosheets, after decorating with group VIII metal promoters (*i.e*., Fe, Co or Ni), convert high-density polyethylene (HDPE) into alkylaromatics and olefins at low temperature and ambient pressure without using any solvent or hydrogen: 2D Ni/WO_3_ with abundant Brønsted acidic sites initiates HDPE cracking at a low temperature of 240 °C; 2D Fe/WO_3_ with low energy barrier of cyclization achieves a high HDPE conversion to 84.2% liquid hydrocarbons with a selectivity of 30.9% to aromatics at 300 °C. *In-situ* spectroscopic investigations and supplementary theoretical calculations illustrate that these aromatics are formed through the cyclization of alkene intermediates. These 2D catalysts also display high efficiency in the low-temperature cracking of single-use commercial polyethylene wastes such as packaging bags and bottles. This work has demonstrated the high potential of 2D non-noble metal catalysts in the efficient upcycling of waste polyolefin at mild conditions.

DOI: 10.1002/anie.2024XXXXX

**Table of Contents**

[1. Experimental procedures 3](#_Toc181195057)

[1.1 Chemical materials 3](#_Toc181195058)

[1.2 Catalyst preparation 3](#_Toc181195059)

[1.3 Catalyst characterization 3](#_Toc181195060)

[1.4 Catalytic performance test and statistical analysis 4](#_Toc181195061)

[1.5 *In situ* spectroscopy investigation of HDPE catalytic cracking 4](#_Toc181195062)

[1.6 Density functional calculations 5](#_Toc181195063)

[2. Results and discussion 6](#_Toc181195064)

[2.1 ICP-OES data of 2D M/WO_3_ nanosheets 6](#_Toc181195065)

[2.2 Porous analysis of 2D M/WO_3_ nanosheets 6](#_Toc181195066)

[2.3 XPS analysis of 2D M/WO_3_ nanosheets 6](#_Toc181195067)

[2.4 XANES and EXAFS analysis of Co/WO_3_ and Ni/WO_3_ nanosheets 7](#_Toc181195068)

[2.5 Chromatography analysis of 2D M/WO_3_ nanosheets 8](#_Toc181195069)

[2.6 Nuclear magnetic resonance analysis of 2D M/WO_3_ nanosheets 9](#_Toc181195070)

[2.7 Characterizations of 3D bulk Fe/WO_3_ catalyst 10](#_Toc181195071)

[2.8 Chromatography analysis of 2D Pt/WO_3_ nanosheets 10](#_Toc181195072)

[2.9 Optimized structure used for the DFT calculation 11](#_Toc181195073)

[2.10 Reusability performance of 2D Fe/WO_3_ nanosheets 11](#_Toc181195074)

[2.11 *In situ* spectroscopy analysis 14](#_Toc181195075)

[2.12 Acidic analysis of 2D M/WO_3_ nanosheets 14](#_Toc181195076)

[References 15](#_Toc181195077)

[Author Contributions 15](#_Toc181195078)

# Experimental procedures

## Chemical materials

Mg(NO_3_)_2_∙6H_2_O (AR, 99%), Al(NO_3_)_3_∙9H_2_O (AR, 98%) and Na_2_WO_4_·2H_2_O (AR, 99%) were purchased from Aladdin. Hydrochloric acid (AR, 36.5%), ethanol (AR, 99%) and urea (AR, 99%) were purchased from Sinopharm. KBr (AR, 99%), H_2_PtCl_6_·6H_2_O (AR, 99%) was purchased from Sigma Aldrich. Fe(NO_3_)_3_∙9H_2_O, Co(NO_3_)_2_∙6H_2_O and Ni(NO_3_)∙6H_2_O were purchased from Macklin Inc. The chemicals were used as received without further purification. The high-density polyethylene (HDPE) substrates (*M*w~66000Da) were purchased from Zhong Xin Inc. The HDPE plastic bottle was obtained from Shui Shan plastic products Inc., the HDPE packaging was obtained from Sam’s Club, the low-density polyethylene (LDPE) packaging was obtained from Zhong Xin Inc., and the LDPE dropper was obtained from Gao Yan Inc. Distilled deionized (DDI) water was obtained from a Milli-Q Hx7150 water purifier system.

## Catalyst preparation

The 2D M/WO_3_ (M=Fe, Co, Ni and Pt) catalysts were prepared by an impregnation method, for which the WO_3_ nanosheets were prepared by a space-confined method we reported previously.^[1]^ Briefly, Mg-Al-CO_3_ layered double hydroxide (LDH) was first synthesized via a hydrothermal method at 90 °C for 24 h. The LDH-WO_4_^2−^ composite was synthesized via a structure-reconstruction route and following calcinated at 500 °C for 2 h to obtain the LDO-WO_3_ composite. Next, 2D WO_3_ nanosheets were obtained by etching LDO in the HCl solution and further calcination at 300 °C for 2 h to remove crystalline water. Subsequently, 100 mg of 2D WO_3_ was dispersed in deionized water, and 0.5 mL Fe(NO_3_)_3_∙9H_2_O, Co(NO_3_)_2_∙6H_2_O, Ni(NO_3_)∙6H_2_O and H_2_PtCl_6_·6H_2_O aqueous solution (1 mg∙mL^-1^) was added into the mixture under vigorous stirring for 12 h and following by filtration and washing. 2D M/WO_3_ catalyst was obtained by annealing at 300 °C for 1 h in the reductive atmosphere of Ar/H_2_ (5% H_2_).

## Catalyst characterization

Inductively Coupled Plasma Optical Emission Spectrometry (ICP-OES). ICP-OES was operated on an Agilent 5110 and the catalysts was dissolved in a mixture of aqua regia and hydrofluoric acid by boiling at 200 °C for 1 h.

Atomic Force Microscope (AFM) . AFM was operated on a Nanoscope IIIa Multimode AFM in tapping mode using a Si tip cantilever with a force constant of 40 N·m^−1^ and the catalyst was deposited on a clean silicon wafer.

Electron Microscopy. Atomic-resolution scanning transmission electron microscopy (STEM) images were recorded on a JEOL ARM200F operated at 200 kV with cold field emission gun and double hexapole Cs correctors. STEM images were recorded using a high-angle annular dark-field (HAADF) detector with convergence angle of 30 mrad and collection angle between 90 and 370 mrad. The Hitachi SU8010 scanning electron microscope (SEM) was operated at an accelerating voltage of 3 kV to obtain the morphology and composition.

X-ray Diffraction. Powder X-ray diffraction (PXRD) patterns were performed on a Bruker D8 Advance system using Cu Kα radiation (*λ* = 1.54178 Å).

Nitrogen Adsorption. N_2_ physisorption experiments, Brunauer-Emmett-Teller (BET) surface area analysis, and Barrett-Joyner-Halenda (BJH) mesopore size analysis were conducted using a Micromeritics APSP 2460 surface characterization analyzer. Typically, 0.1 g material was outgassed in vacuum at 200 °C before N_2_ adsorption at -196 °C.

X-ray Photoelectron Spectrometer. The catalyst surface was analyzed by a Thermo Scientific ESCALAB 250 Xi X-ray photoelectron spectrometer (XPS) to investigate the surface composition and oxygen species. The binding energy was calibrated to C 1s at 284.8 eV.

X-ray absorption spectra (XANES and EXAFS) were measured at the BL14W1 beamline of Shanghai Synchrotron Radiation Facility (SSRF), which operates at 3.5 GeV in “top-up” mode with injection current of 220 mA. The Fe,Co and Ni *K*-edge XANES data were recorded in a fluorescence mode. Fe foil (99.99%), FeO (99.5%, Alfa Aesar), Fe_2_O_3_ (99.5%, Alfa Aesar), Co foil (99.99%), CoO (99.5%, Alfa Aesar), Co_3_O_4_ (99.5%, Alfa Aesar), Ni foil (99.99%) and NiO (99.5%, Alfa Aesar) were used as references.

Ammonia Temperature Programmed Desorption (NH_3_-TPD). The NH_3_-TPD was performed in a BELCAT II with TCD detector. 0.1 g catalyst was loaded in the fixed bed reactor, then heated up to 300 °C in 100 mL∙min^-1^ He and cooled to 50 °C. The NH_3_ adsorption was performed by feeding NH_3_/He (10 vol.% NH_3_ in He). The NH_3_-TPD measurement was performed in 100 mL∙min^-1^ He from 50 °C to 700 °C at a heating rate of 10 °C min^-1^.

Pyridine Infrared Spectroscopy. Pyridine-IR was conducted by a Thermo Fisher Nicolet iS20 Fourier transform infrared spectrometer. The pretreatment was carried out in vacuum at 250 °C for 1 h. The adsorption of pyridine vapor was performed at room temperature, and subsequently purged with He. Pyridine-IR spectra were taken at 250 °C with a collection of 32 times at a resolution of 4 cm^-1^.

^31^P solid-state Nuclear Magnetic Resonance (NMR). The catalysts were outgassed at 150 °C under vacuum for 4 h while 100 mg Trimethylphosphine oxide (TMPO) was dissolved in 30 mL dichloromethane under Ar atmosphere. Then 50 mg catalysts were added into the TMPO solution under 1 h of ultrasonication. The solids were centrifuged and dried at room temperature for 24 h to remove the solvent. The ^31^P solid-state NMR experiments were performed on a Bruker AVANCE III HD 600 MHz spectrometer. Magic angle spinning (MAS) experiments were performed on 3.2 mm MAS probes at a spinning rate of 15 kHz. The ^31^P signals were referenced to that of NH_4_H_2_PO_4_ at 0.81 ppm.

## Catalytic performance test and statistical analysis

The activity test was performed in Parr autoclave modified with gas inlet and outlet connector and flow regulating valves. The plastic (HDPE, *Mw*~66000Da) was mixed with catalyst at a weight ratio of 1:1 in a 50 mL stainless-steel reactor with mechanical stirring. It was purged with N_2_ for 1 h before reaction. The HDPE and catalyst were heated up, and reacted for certain hours in the sealed reactor.

After 12 hours at 300 °C, the liquid/wax products were recovered for characterization by dissolving in hot methylene chloride (CH_2_Cl_2_). The residual oil was then separated by centrifuging. The products of HDPE were analyzed by an Agilent 7890A gas chromatography (GC) equipped with an 5977B inert mass spectroscopy detector (MSD) using a capillary column (Agilent J&W HP-5ms, (5%-phenyl)-methylpolysiloxane, 30 m, 0.32 mm, 0.25 μm). Alkanes, alkenes and aromatics mixture standard samples were used as external standard to identify and quantify the liquid products. Figure S1 presents the GC-MS spectra of standard samples containing 500 μg∙mL^-1^ normal C8-C40 alkanes, 500 μg∙mL^-1^ benzene, toluene, ethylbenzene, acenaphthene, benz-anthracene, chrysene, fluoranthene, fluorene, 1-methylnaphthalene, 2-methylnaphthalene, naphthalene, phenanthrene, pyrene and 500 ug∙mL^-1^ C7-C18 alkenes. At the same time, the residual solid was collected and then dissolved in toluene at high temperature to remove unreacted PE.


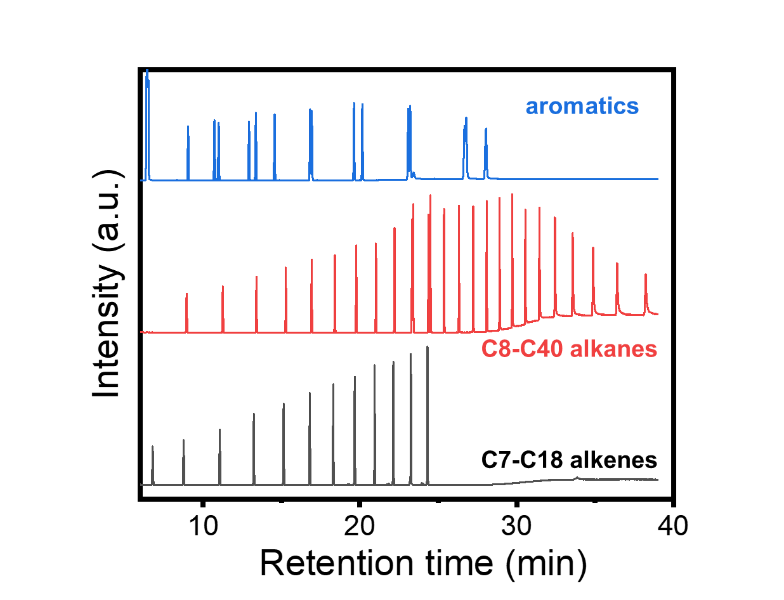


**Figure S1.** GC-MS spectra of liquid standard dissolved in CH_2_Cl_2_.

The mass balance was carried out by weighing the HDPE residue and liquid products as indicated in equation: m_HDPE_ = m_gas_ + m_liquid_ +m_residue_, where m_HDPE_, m_gas_, m_liquid_, and m_residue_ refer to the mass of initial PE, volatile products, liquid products, carbon disposition and unreacted PE, respectively. The weight differences between unreacted HDPE and total HDPE indicated a quantitative conversion. The m_unreacted PE_ was determined by taking the subtraction of masses of the residue and carbon deposition products. Conversion of HDPE is calculated as:

Conversion of HDPE = $\left\{1-\frac{mass\text{ }of\text{ unreacted }HDPE}{\text{mass of initial }HDPE}\right\}\times100\%$

Yields of gaseous products, liquid products and solid products are defined as:

Yield = $\left\{\frac{mass\text{ of products}}{mass\text{ of initial HDPE}}\right\}\times100\%$

The CH_2_Cl_2_ in liquid products were removed and spectra of the products were recorded in 1,1,2,2-tetrachloroethane-d_2_ (TCE-d_2_). ^1^H NMR spectra were acquired at Bruker AVANCE III 600 MHz spectrometer, and were analyzed using MestReNova. ^13^C NMR spectra were acquired on a Bruker AVANCE III 500 MHz spectrometer. A Bruker Cryoprobe Prodigy 5mm BBO was used to enhance sensitivity for direct ^13^C detection. ^13^C spectra were recorded using a long relaxation delay (10 s) to ensure quantitative intensities. Chemical shifts (*δ*, ppm) were calibrated using the residual proton signals of the solvent and referenced to tetramethylsilane (TMS).

The matrix-assisted laser desorption ionization time-of-ﬂight mass spectrum (MALDI-TOF-MS, cation mode) of products was obtained on a Bruker rapiflex MALDI Tissuetyper, using methylene chloride (CH_2_Cl_2_) as solvent and trans-2-[3-(4-tert-Butylphenyl)-2-methyl-2-propenylidene] malononitrile (DCTB) as matrix.

## *In situ* spectroscopy investigation of HDPE catalytic cracking

*In-situ* DRIFTS spectroscopy*.* Diffuse reflectance infrared Fourier transform (DRIFT) experiments were performed on a Nicolet iS50 FTIR spectrometer, equipped with *in-situ* diffuse reflectance cell (Harrick) and MCT detector cooled by liquid N_2_. The spectra were collected at a resolution of 4 cm^-1^ and 32 scans. The pretreatment was carried out by loading sample (0.04 g) in the cell and heating to 200 °C for 1 h in N_2_, followed by cooling to 30 °C. The background spectrum was collected at 30 °C. For the reaction of catalytic cracking of octane, the catalysts were exposed to 100 ppm octane then heated to 300 °C at a heating rate of 5 °C∙min^-1^ for 1 h.

*In-situ* Raman spectroscopy. The micro-Raman spectra were obtained by a Horiba LabRAM Odyssey micro-Raman spectrometer with a LEICA DMLM microscope equipped with *in-situ* cell (Linkam) using a 532 nm Ar laser light source and an acquisition time of 30 s for 4 scans.

## Density functional calculations

The Vienna Ab Initio Package (VASP) was employed to perform all the density functional theory (DFT) calculations within the generalized gradient approximation (GGA) using the Perdew, Burke, and Enzerhof (PBE) formulation.^[1, 2]^ The projected augmented wave (PAW) potentials were applied to describe the ionic cores and take valence electrons into account using a plane wave basis set with a kinetic energy cutoff of 450 Ev.^[3, 4]^ Partial occupancies of the Kohn–Sham orbitals were allowed using the Gaussian smearing method and a width of 0.1 eV. The electronic energy was considered self-consistent when the energy change was smaller than 10^−5^ eV. A geometry optimization was considered convergent when the force change was smaller than 0.05 eV/Å. Grimme’s DFT-D3 methodology was used to describe the dispersion interactions.^[5]^ The vacuum spacing perpendicular to the plane of the structure is 20 Å. Finally, the adsorption energies (E_ads_) were calculated as E_ads_= E_ad/sub_ -E_ad_ -E_sub_, where E_ad/sub_, E_ad_, and E_sub_ are the total energies of the optimized adsorbate/substrate system, the adsorbate in the structure, and the clean substrate, respectively. The free energy was calculated using the equation:

G = E_ads_ + ZPE - TS

where G, E_ads_, ZPE and TS are the free energy, total energy from DFT calculations, zero-point energy and entropic contributions, respectively. Transition states for elementary reaction steps were determined by the nudged elastic band (NEB) method. In the NEB method, the path between the reactant and product is discretized into a series of structural images.

# Results and discussion

## ICP-OES data of 2D M/WO_3_ nanosheets

**Table S1.** ICP-OES results of Fe/WO_3_, Co/WO_3_ and Ni/WO_3_ nanosheets.

| Sample | Weight (g) | Solution volume (mL) | Metal content in the solution (mg·L^-1^) | Metal loading (mg·g^-1^) |
| --- | --- | --- | --- | --- |
| Fe/WO_3_ | 0.0312 | 10 | 25.99 | 8.33 |
| Co/WO_3_ | 0.0343 | 10 | 27.85 | 8.12 |
| Ni/WO_3_ | 0.0308 | 10 | 25.44 | 8.26 |

## Porous analysis of 2D M/WO_3_ nanosheets

**Table S2.** N_2_ physisorption data of Fe/WO_3_, Co/WO_3_ and Ni/WO_3_ nanosheets.

| Sample | BET surface area (m^2^∙g^-1^) | Pore volume (cm^3^∙g^-1^) | BJH pore size (nm) |
| --- | --- | --- | --- |
| Fe/WO_3_ | 43.1 | 0.30 | 27.6 |
| Co/WO_3_ | 43.8 | 0.27 | 30.2 |
| Ni/WO_3_ | 43.5 | 0.29 | 30.0 |


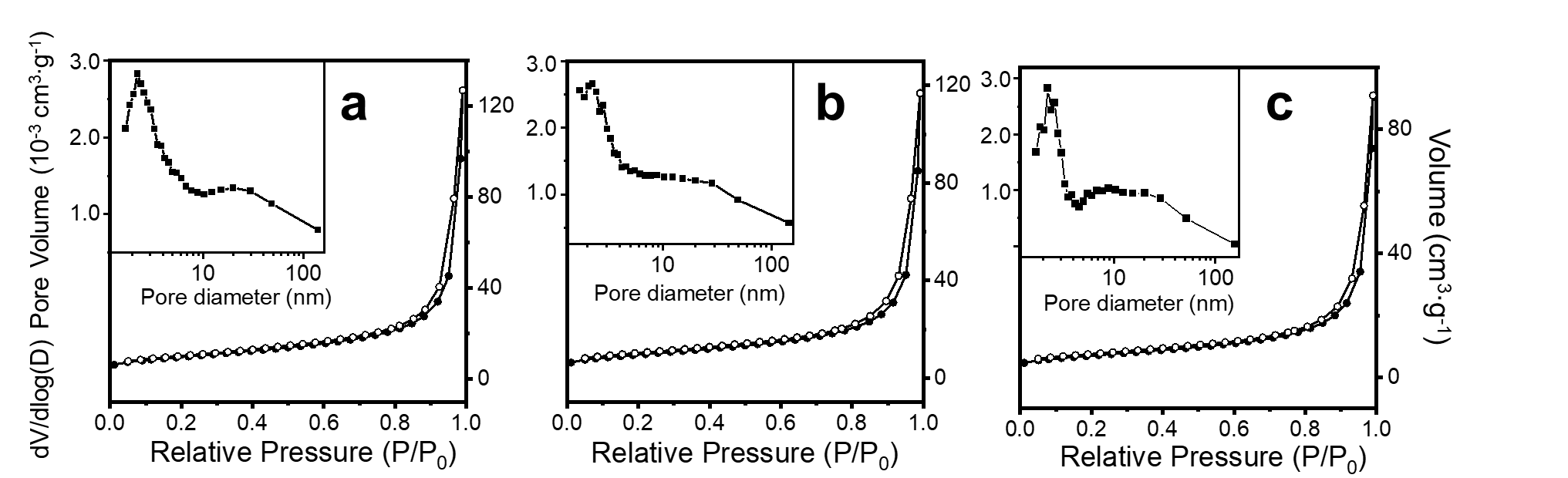


**Figure S2.** N_2_ adsorption-desorption isotherms and pore size distribution of (a) Fe/WO_3_, (b) Co/WO_3_ and (c) Ni/WO_3_ nanosheets.

## XPS analysis of 2D M/WO_3_ nanosheets

**Table S3.** XPS data of Fe/WO_3_, Co/WO_3_ and Ni/WO_3_.

| **Sample** | **Fe 2*p*** | | **Co 2*p*** | | **Ni 2*p*** | | **W 4*f*** | |
| --- | --- | --- | --- | --- | --- | --- | --- | --- |
|  | Fe^3+^(eV)  (R_at_,%) | Fe^2+^(eV)  (R_at_,%) | Co^3+^(eV)  (R_at_,%) | Co^2+^(eV)  (R_at_,%) | Ni^3+^(eV)  (R_at_,%) | Ni^2+^(eV)  (R_at_,%) | W^5+^(eV)  (R_at_,%) | W^6+^(eV)  (R_at_,%) |
| Fe/WO_3_ | 727.4/714.6  (28.5%) | 722.6/710.8  (71.5%) | - | - | - | - | 36.1/34.0  (15.4%) | 37.1/35.0  (84.6%) |
| Co/WO_3_ | - | - | 797.2/782.2  (56.5%) | 795.2/780.2  (43.5%) | - | - | 36.1/34.0  (21.4%) | 37.1/35.0  (78.6%) |
| Ni/WO_3_ | - | - | - | - | 874.4/856.8  (58.7%) | 873.2/855.6  (41.3%) | 36.1/34.0  (20.2%) | 37.1/35.0  (79.8%) |


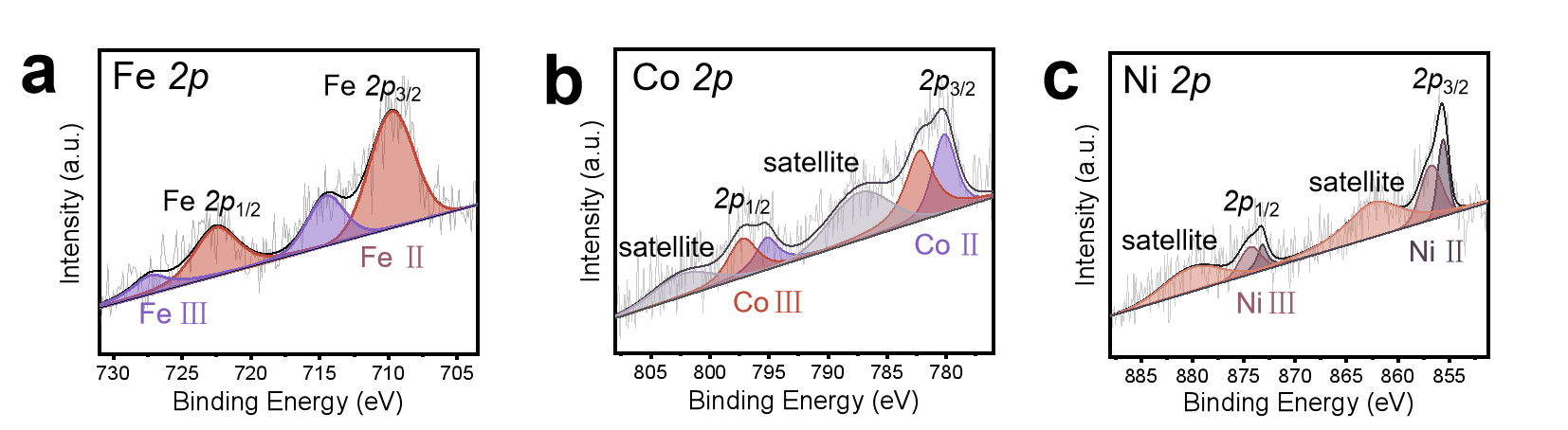


**Figure S3.** XPS data of Fe *2p* and Co *2p* and Ni *2p* for (a) Fe/WO_3_, (b) Co/WO_3_ and (c) Ni/WO_3_ nanosheets.

## XANES and EXAFS analysis of Co/WO_3_ and Ni/WO_3_ nanosheets


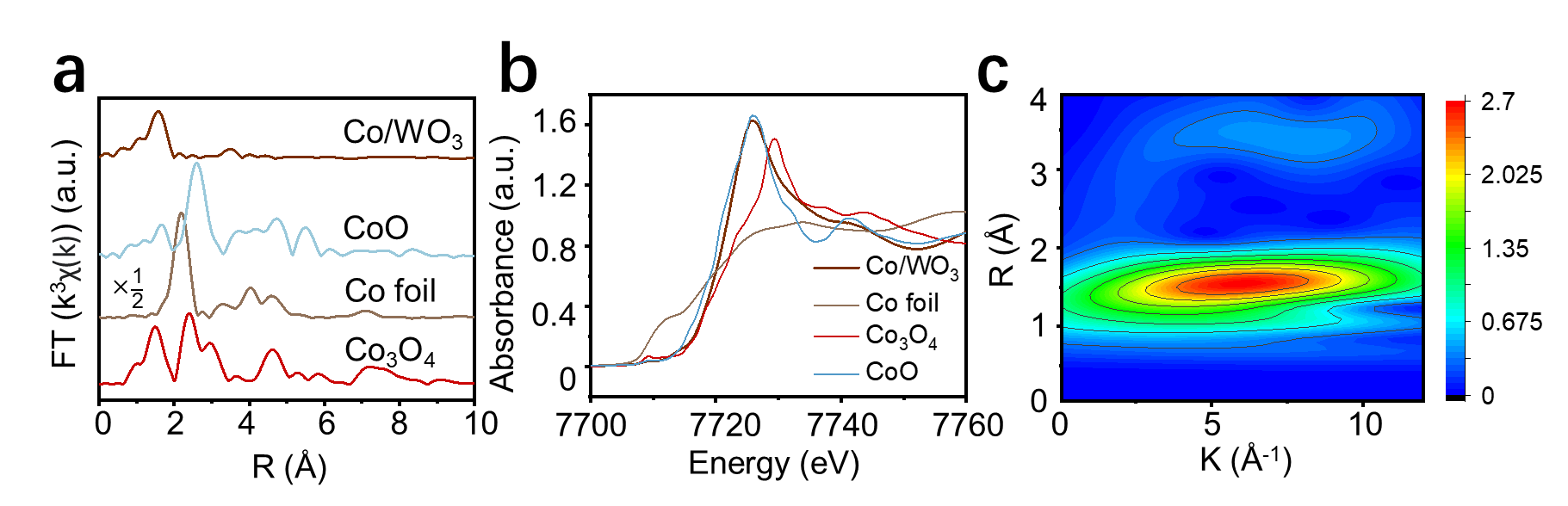


**Figure S4.** (a) The *k*^3^-weighted Fourier transform spectra from EXAFS, (b) The normalized XANES spectra at the Co *K* edge of Co/WO_3_, Co foil, CoO and Co_3_O_4_, (c) WT-EXAFS plots of Co/WO_3_.


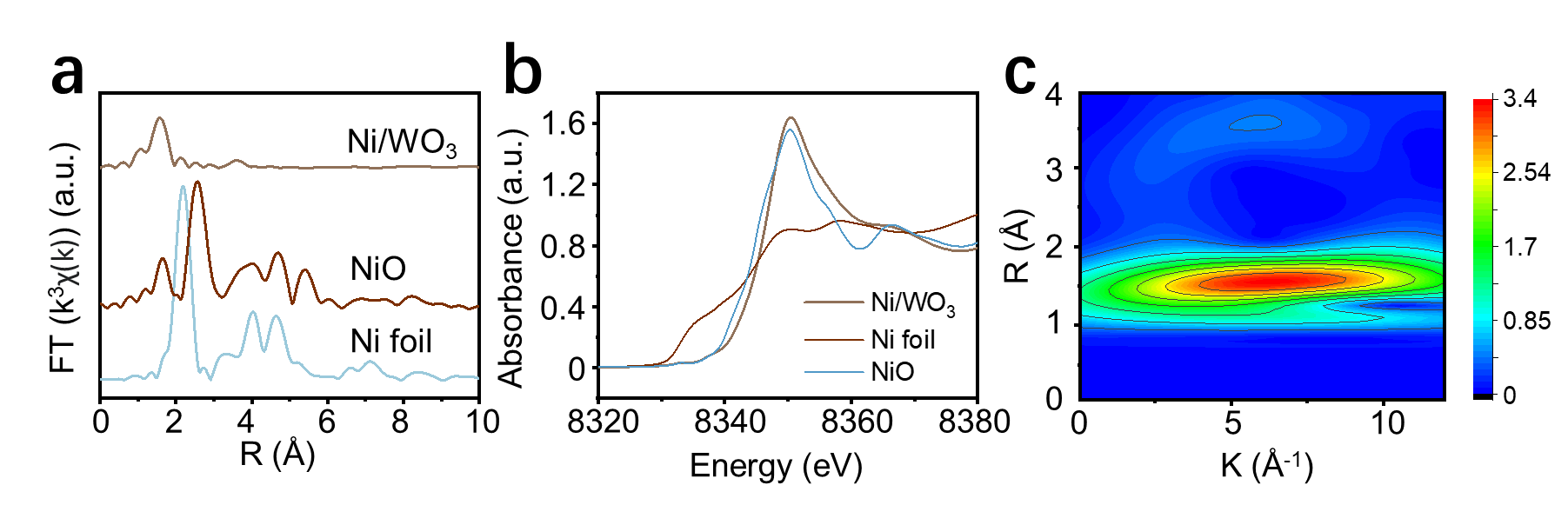


**Figure S5.** (a) The *k*^3^-weighted Fourier transform spectra from EXAFS, (b) The normalized XANES spectra at the Ni *K* edge of Ni/WO_3_, Ni foil, NiO, (c) WT-EXAFS plots of Ni/WO_3_.

## Chromatography analysis of 2D M/WO_3_ nanosheets


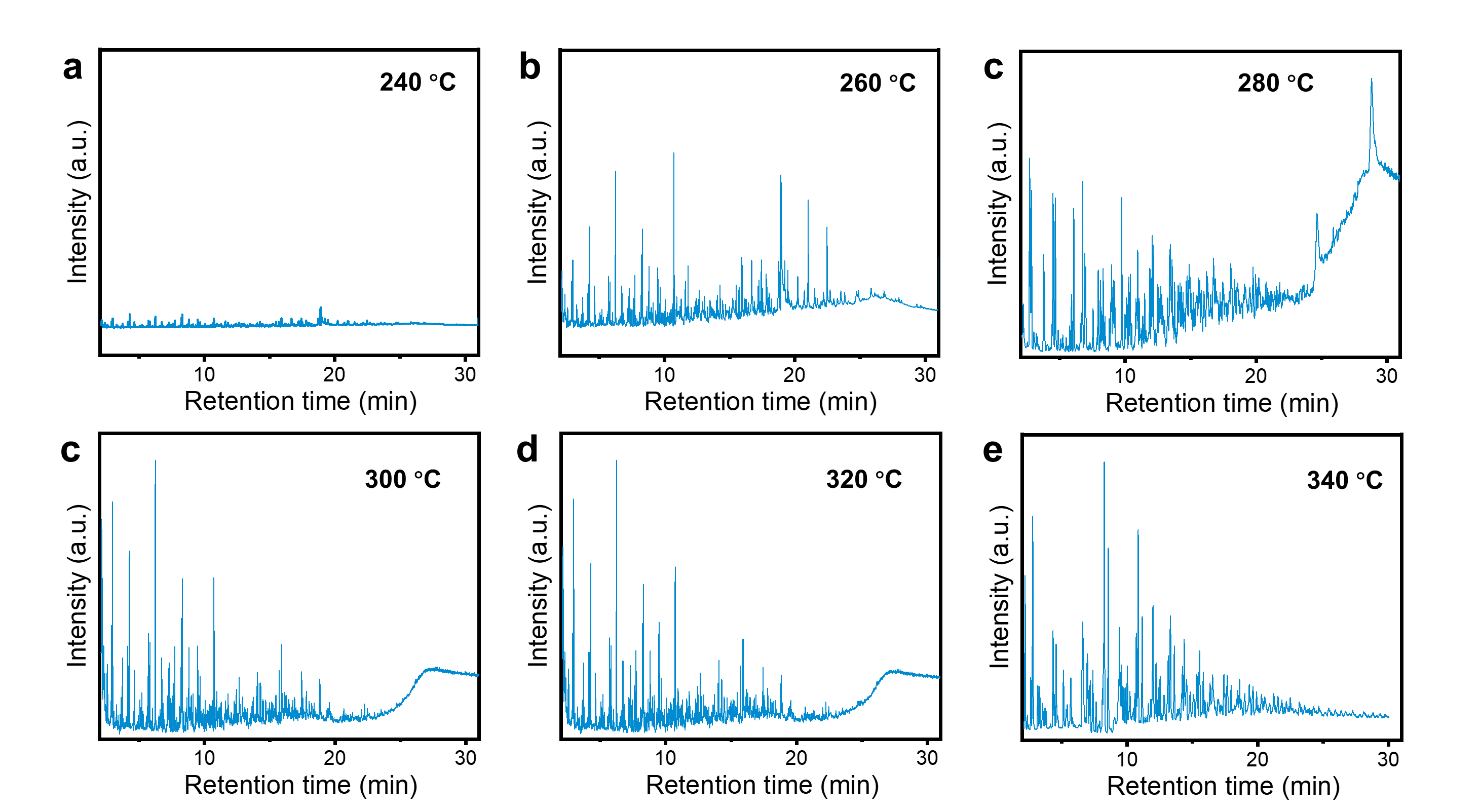


**Figure S6.** GC pattern of liquid products obtained for HDPE cracking over 2D Fe/WO_3_ nanosheets at different temperatures.


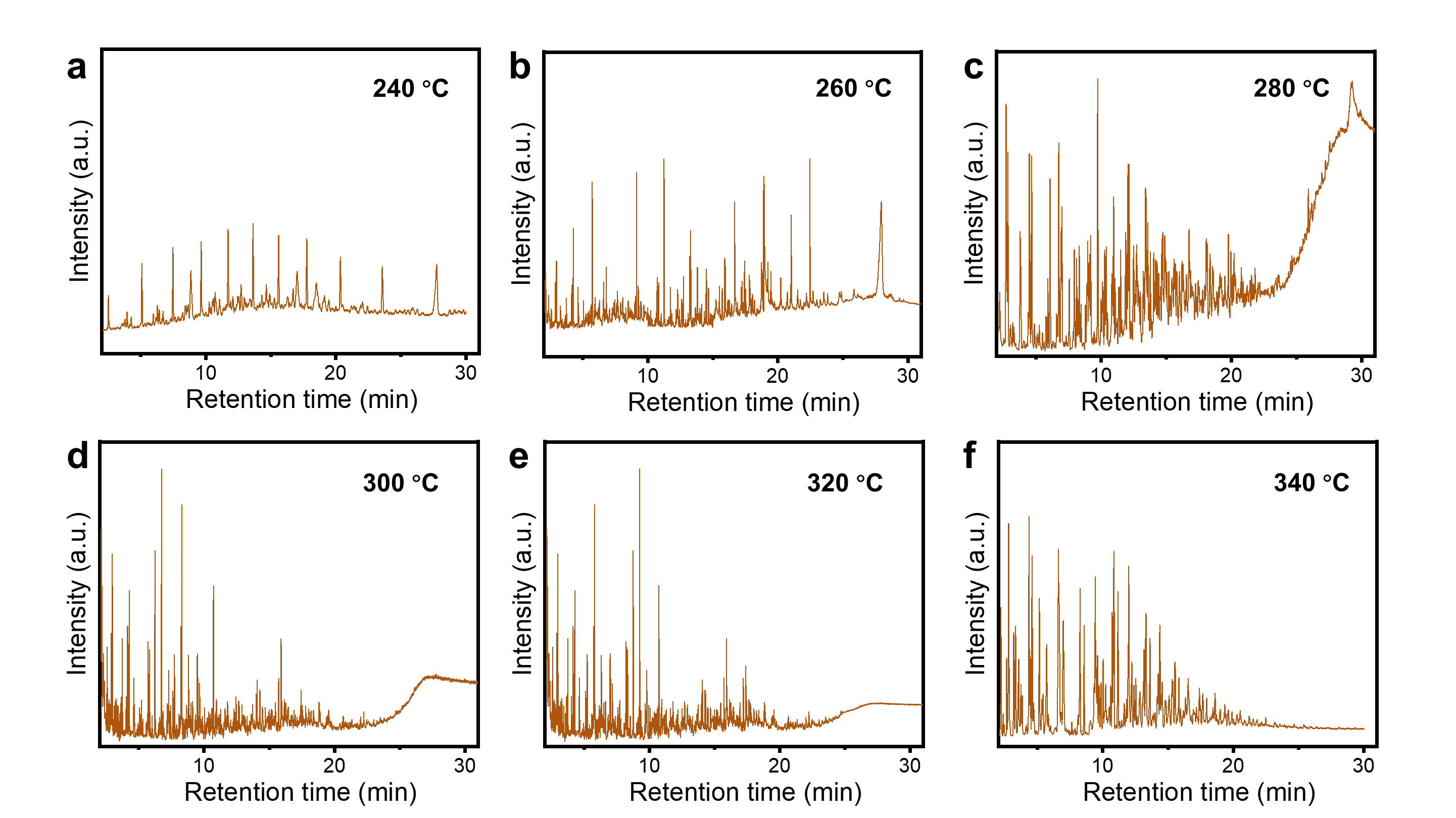


**Figure S7.** GC pattern of liquid products obtained for HDPE cracking over 2D Co/WO_3_ nanosheets at different temperatures.


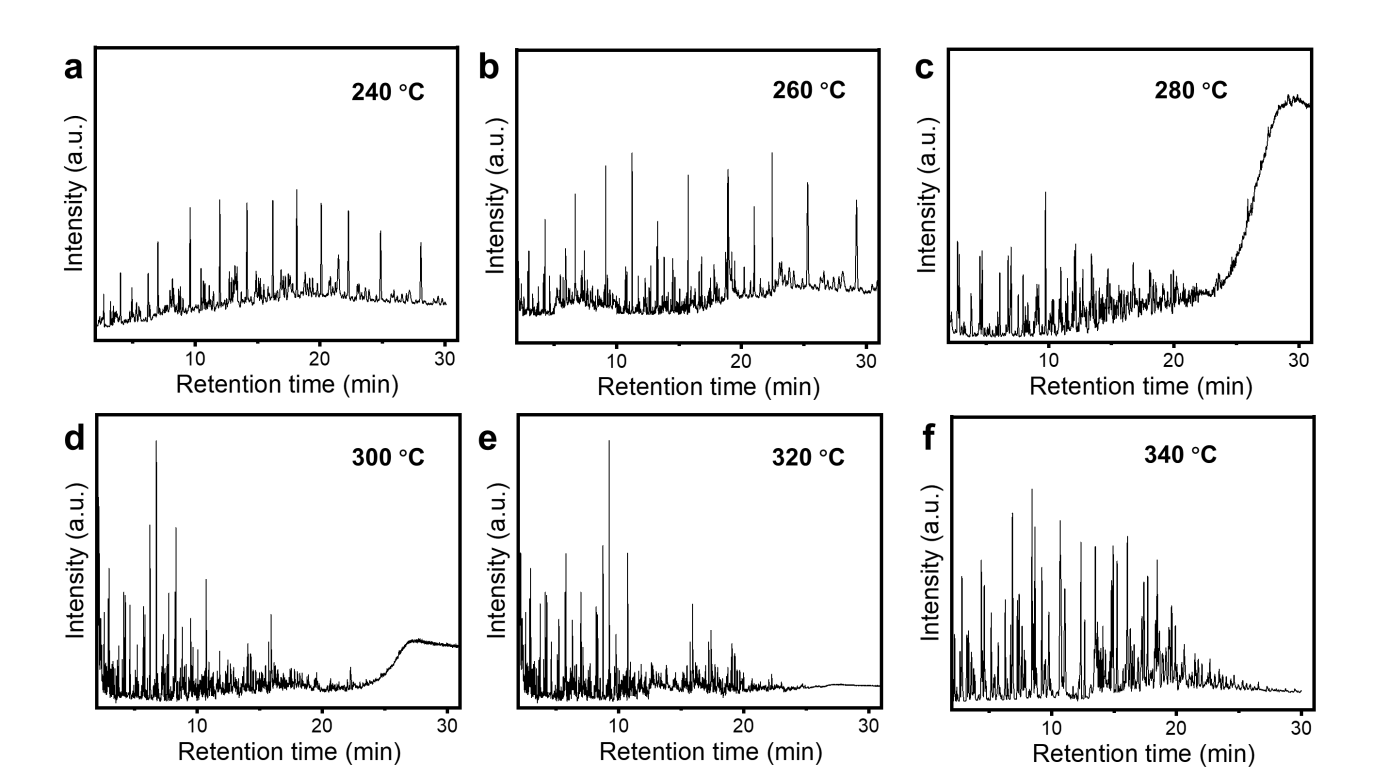


**Figure S8.** GC pattern of liquid products obtained for HDPE cracking over 2D Ni/WO_3_ nanosheets at different temperatures.

## Nuclear magnetic resonance analysis of 2D M/WO_3_ nanosheets

**
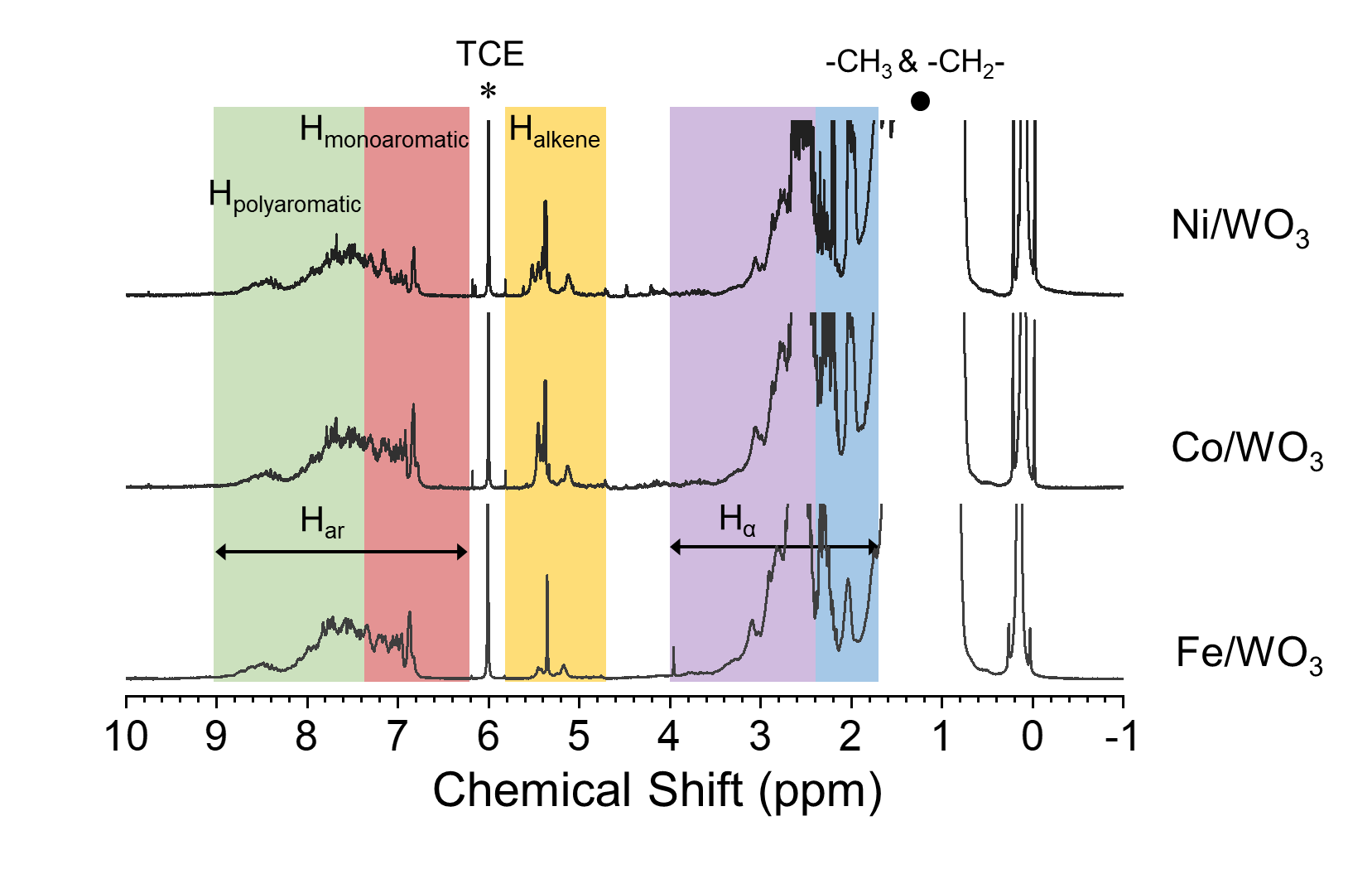
**

**Figure S9.** ^1^H NMR spectra of the soluble component recovered from the solid residue for HDPE cracking over Fe/WO_3_, Co/WO_3_ and Ni/WO_3_ nanosheets. [* Indicates residual protons in the solvent (TCE-d_2_).]


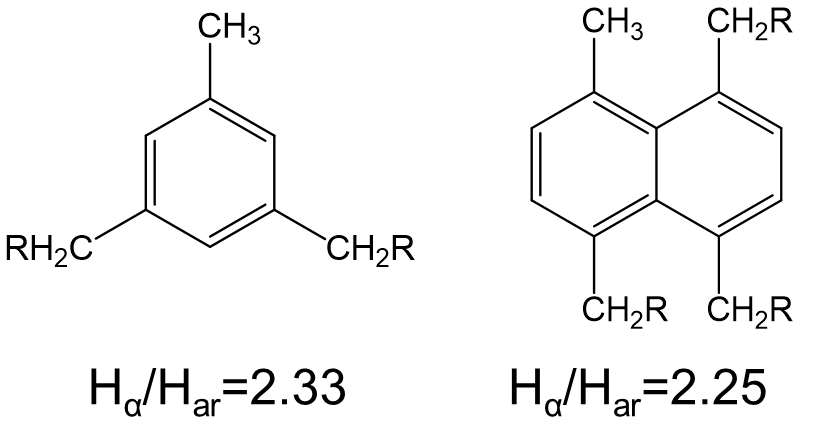


**Figure S10.** Possible structures for substituted alkylbenzenes, and their corresponding H_α_/H_ar_ ratios.

**Table S4.** Comparison of polyolefin catalytic cracking and product distribution in this work and in the literature.

| Catalyst | Atmosphere | Plastic | T/°C | t/h | Conversion/% | Yield on carbon basis/% | | | Aromatic yield/% | Olefin yield/% | Ref. |
| --- | --- | --- | --- | --- | --- | --- | --- | --- | --- | --- | --- |
|  |  |  |  |  |  | Gas | Liquid (selectivity to aromatic) | Residue |  |  |  |
| 0.8wt.%Fe/WO_3_ nanosheets | N_2_ | HDPE(*Mw*, 66000) | 300 | 12 | 94.9 | 10.7 | 84.2(30.9%) | 5.1 | 26.0 | 16.8 | This work |
| 0.8wt.%Co/WO_3_ nanosheets | N_2_ | HDPE(*Mw*, 66000) | 300 | 12 | 94.0 | 10.4 | 83.6(28.7%) | 6.0 | 24.0 | 21.0 |  |
| 0.8wt.%Ni/WO_3_ nanosheets | N_2_ | HDPE(*Mw*, 66000) | 300 | 12 | 94.5 | 10.5 | 84.0(26.5%) | 5.5 | 22.3 | 23.5 |  |
| WO_3_ nanosheets | N_2_ | HDPE(*Mw*, 66000) | 300 | 12 | 7.0 | 11.3 | 78.7(20.0%) | 10.0 | 15.7 | 41.3 |  |
| 0.8wt.%Fe/WO_3_ (bulk) | N_~~2~~_ | HDPE(*Mw*, 66000) | 300 | 12 | 44.0 | 12.6 | 31.4(6.1%) | 56.0 | 1.9 | 12.6 |  |
| 0.2wt.%Pt/WO_3_ nanosheets | N_2_ | HDPE(*Mw*, 66000) | 300 | 12 | 99.5 | 40.3 | 59.2(0.2%) | 0.5 | 0.1 | 23.3 |  |
| 0.8wt.%Ni/WO_3_ nanosheets | N_2_ | HDPE(*Mw*, 66000) | 240 | 12 | 25.6 | 0 | 25.6(0.4%) | 74.4 | 0.1 | 5.6 |  |
| Catalyst | Atmosphere | Plastic | T/°C | t/h | Conversion/% | Yield on carbon basis/% | | | Aromatic yield/% | Olefin yield/% | Ref. |
|  |  |  |  |  |  | Gas | Liquid (selectivity to aromatic) | Coke |  |  |  |
| USY | N_2_ | PE | 500 | 0.5 | 99.1 | 42.6 | 56.5(22.4%) | 0.9 | 12.66 | 9.27 | ^[2]^ |
| 8wt.%Fe(O-550)/USY | N_2_ | PE | 500 | 0.5 | 98.6 | 26.1 | 72.5(24.7%) | 1.4 | 17.91 | 11.96 |  |
| Catalyst | Atmosphere | Plastic | T/°C | t/h | Conversion/% | Yield on carbon basis/% | | | Aromatic yield/% | Olefin yield/% | Ref. |
|  |  |  |  |  |  | C1-4 | C5+ Paraffin | Aromatic |  |  |  |
| ZSM-5 | 67%N_2_, 29.7%Ar, 3.3%H_2_ | PE | 400 | 4 | 100 | 32 | 40 | 28(83%methylated aro.) | 28.00 | - | ^[3]^ |
| Zn/ZSM-5 | 67%N_2_, 29.7%Ar, 3.3%H_2_ | PE | 400 | 4 | 100 | 38 | 30 | 32(86%methylated aro.) | 32.00 | - |  |
| Catalyst | Atmosphere | Plastic | T/°C | t/h | Conversion/% | Yield on carbon basis/% | | | Aromatic yield/% | Olefin yield/% | Ref. |
|  |  |  |  |  |  | Gas | Liquid | Coke |  |  |  |
| TiO_2_ | N_2_ | LDPE | 300 | 0.5 | 76 | 8 | 85 | 7 | - | - | ^[5]^ |
| 2.5wt.%Cu/TiO_2_ | N_2_ | LDPE | 500 | 0.5 | 83 | 2.5 | 95 | 2.5 | - | - |  |
| Catalyst | Atmosphere | Plastic | T/°C | t/h | Conversion/% | Yield on carbon basis/% | | | Aromatic yield/% | Olefin yield/% | Ref. |
|  |  |  |  |  |  | Gas | Liquid | Coke |  |  |  |
| FCC | N_2_ | LDPE | 450 | 1 | 62.63 | 31.17 | 31.46 | 37.37 | - | - | ^[6]^ |
| 5wt.%Cu/FCC | N_2_ | LDPE | 450 | 1 | 69.2 | 34.28 | 34.92 | 30.8 | - | - |  |
| Catalyst | Atmosphere | Plastic | T/°C | t/h | Conversion/% | Yield on carbon basis/% | | | Aromatic yield/% | Olefin yield/% | Ref. |
|  |  |  |  |  |  | Gas | Liquid (selectivity to BETX) | Coke |  |  |  |
| HZSM-5 | CO_2_ | LDPE | 550 | - | 95.12 | 31.95 | 63.17(34%) | 4.88 | 31.59 | - | ^[7]^ |
| 3wt.%Fe/HZSM-5 | CO_2_ | LDPE | 550 | - | 99.02 | 32.65 | 66.37(29%) | 0.98 | 24.56 | - |  |
| 3wt.%Ni/HZSM-5 | CO_2_ | LDPE | 550 | - | 99.32 | 28.15 | 71.17(44%) | 0.68 | 41.28 | - |  |
| 3wt.%Co/HZSM-5 | CO_2_ | LDPE | 550 | - | 98.88 | 21.42 | 77.46(28%) | 1.12 | 26.34 | - |  |
| Catalyst | Atmosphere | Plastic | T/°C | t/h | Conversion/% | Yield on carbon basis/% | | | Aromatic yield/% | Olefin yield/% | Ref. |
|  |  |  |  |  |  | Gas | Liquid (selectivity to aromatic) | Carbon |  |  |  |
| Y | N_2_ | HDPE | 600 | - | 81 | 36 | 45(78%) | 10 | 35.10 | - | ^[8]^ |
| 1wt.%Ni/Y | N_2_ | HDPE | 600 | - | 72 | 36 | 36(84%) | 22 | 30.24 | - |  |
| 1wt.%Mo/Y | N_2_ | HDPE | 600 | - | 72 | 38 | 34(84%) | 24 | 28.56 | - |  |
| 1wt.%Fe/Y | N_2_ | HDPE | 600 | - | 73 | 33 | 40(94%) | 14 | 37.6 | - |  |
| 1wt.%Ga/Y | N_2_ | HDPE | 600 | - | 73 | 37 | 36(92%) | 18 | 33.12 | - |  |
| 1wt.%Ru/Y | N_2_ | HDPE | 600 | - | 76 | 42 | 34(95%) | 18 | 32.3 | - |  |
| 1wt.%Co/Y | N_2_ | HDPE | 600 | - | 72 | 36 | 36(79%) | 22 | 28.44 | - |  |

## Characterizations of 3D bulk Fe/WO_3_ catalyst


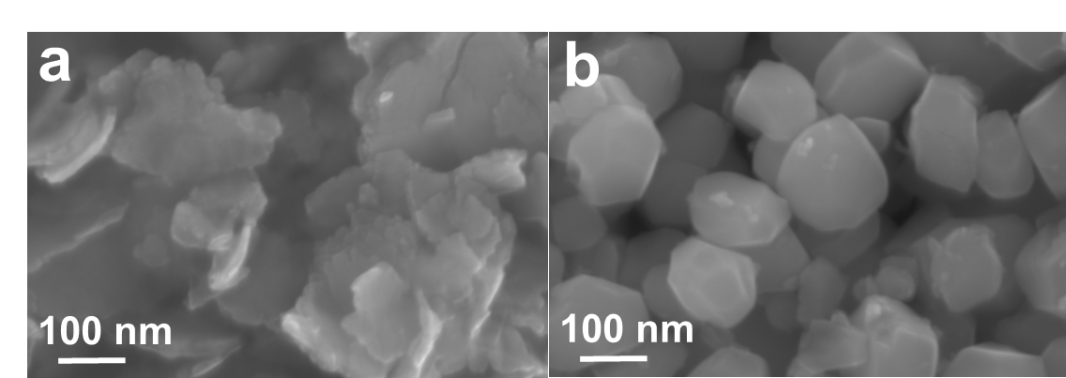


**Figure S11.** SEM images of (a) 2D Fe/WO_3_ and (b) 3D Fe/WO_3_.

**Table S5.** N_2_ physisorption data and acidic analysis of 2D WO_3_ and 3D WO_3_.^[1]^

| Sample | BET surface area  (m^2^∙g^-1^) | Pore volume  (cm^3^∙g^-1^) | BJH pore size  (nm) | Total acidity |
| --- | --- | --- | --- | --- |
| 2D WO_3_ | 48.1 | 0.37 | 31.6 | 1.07 |
| 3D WO_3_ | 8.3 | 0.05 | 18.7 | 0.72 |

## Chromatography analysis of 2D Pt/WO_3_ nanosheets


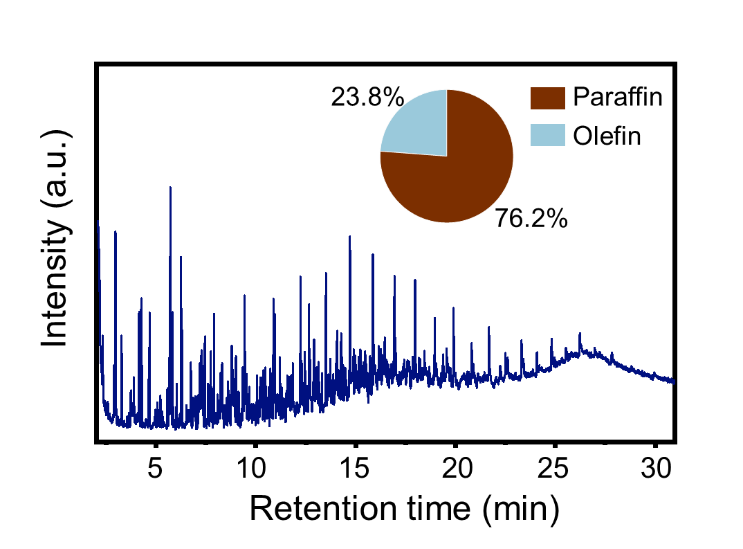


**Figure S12.** GC pattern of liquid products obtained for HDPE cracking over 2D Pt/WO_3_ nanosheets at 300 °C.

## Optimized structure used for the DFT calculation


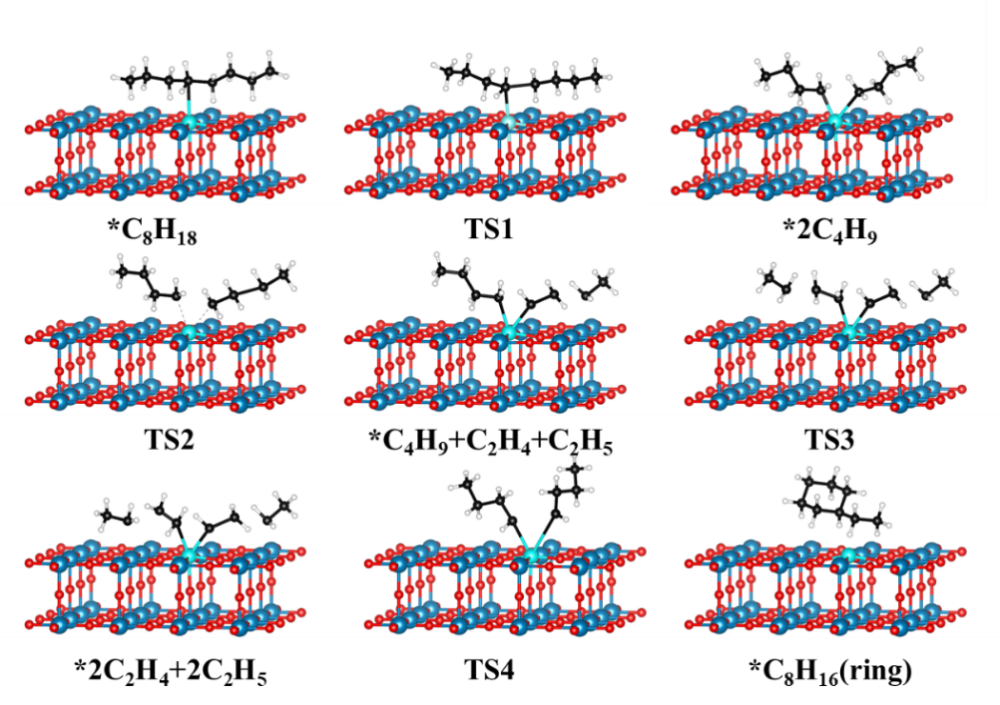


**Figure S13.** The optimized structures used for the theoritical calculation of the catalytic process of octane over Fe/WO_3_ (in Figure 2f).

## Reusability performance of 2D Fe/WO_3_ nanosheets


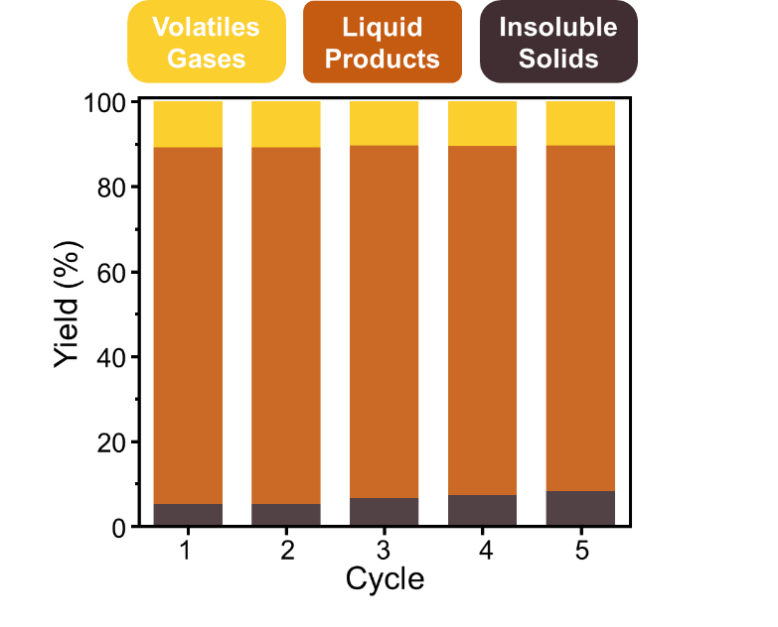


**Figure S14.** The reusability performance of 2D Fe/WO_3_ nanosheets (300 °C, 12 h, N_2_) for 5 cycles.

## *In situ* spectroscopy analysis

**Table S6.** Vibration mode assignments of IR bands on spectra. ^[9,10]^ [Note: ν-stretch, β-in-plane bend]

| Wavenumber (cm^-1^)/  assignment | H_2_O | Aromatic | alkanes | alkenes | alkynes |
| --- | --- | --- | --- | --- | --- |
| 4000-3600 | **√** |  |  |  |  |
| 3180-3000/ν(=C-H) |  | **√** |  |  |  |
| 3000-2850/ν(C-H) |  |  | **√** | **√** | **√** |
| 1470-1450/β(C-H) |  |  | **√** | **√** |  |
| 1370-1350/β(CH_3_) |  |  | **√** |  |  |
| 1680-1640/ν(C=C) |  | **√** |  | **√** |  |
| 2260-2100/ν(C≡C) |  |  |  |  | **√** |

**Table S7.** Vibration mode assignments of Raman bands on spectra. ^[11–13]^ [Note: ν-stretch, β-in-plane bend]

| Assignment | Fe/WO_3_ | Co/WO_3_ | Ni/WO_3_ |
| --- | --- | --- | --- |
| β(W-O-W) | 266 | 266 | 266 |
| ν(W-O-W) _short_ | 697 | 697 | 697 |
| ν(W-O-W) _long_ | 801 | 801 | 801 |
| ν(W=O) | 976 | 976 | 976 |
| ν(C-C) _ring_ | 1590 | 1591 | 1590 |
| β(CH_3_) | 1380 | 1380 | 1380 |
| β(C-C) _ring_ | 1312 | 1312 | 1312 |

## Acidic analysis of 2D M/WO_3_ nanosheets

Acidic properties of catalysts were evaluated by NH_3_-TPD (Figure S15) and pyridine-IR (Table S6, Figure 4b). NH_3_-TPD profile presents physically adsorbed NH_3_ at 128 °C, weakly bonded NH_3_ at 225 °C, and NH_3_ on medium and strong strength acid sites at 300 °C ^[6]^ and 390 °C,^[7]^ respectively. Co/WO_3_ and Ni/WO_3_ exhibit similar NH_3_-TPD profiles, demonstrating that it is WO_3_ that provides abundant acidic sites. As indicated in Figure 4b, the bands at 1540 cm^-1^ and 1490 cm^-1^ were assigned to the Brønsted (B) acid sites.^[8]^ Two absorbance bands at 1450 cm^-1^ and 1610 cm^-1^ assigned to Lewis (L) acid sites.^[9]^

**Table S8.** Acidic content of Fe/WO_3_, Co/WO_3_ and Ni/WO_3_ nanosheets.

| Sample | Acidic properties (μmol·g^-1^) | | |
| --- | --- | --- | --- |
|  | Lewis acid sites (L) | Brønsted acid sites (B) | The ratio of Lewis acidic sites to Brønsted acidic sites (L/B ratio) |
| Fe/WO_3_ | 0.70 | 0.07 | 10 |
| Co/WO_3_ | 0.75 | 0.10 | 7.5 |
| Ni/WO_3_ | 0.72 | 0.11 | 6.5 |


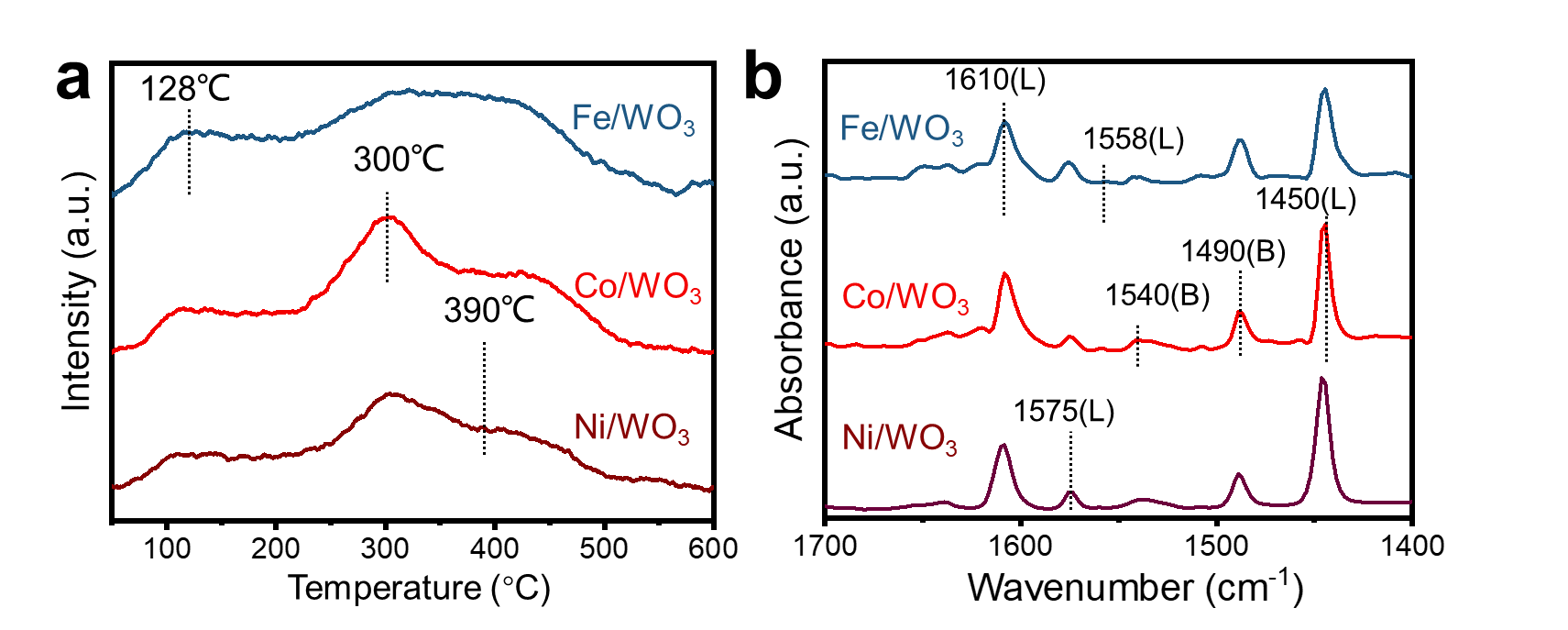


**Figure S15.** (a) NH_3_-TPD profiles of Fe/WO_3_, Co/WO_3_ and Ni/WO_3_.

# References

[1] Q. Zhou, D. Wang, Q. Wang, K. He, K. H. Lim, X. Yang, W.-J. Wang, B.-G. Li, P. Liu, *Angew. Chem. Int. Ed.* **2023**, *62*, e202305644.

[2] Z. Chen, B. J. Erwin, L. Che, *Fuel* **2024**, *363*, 131007.

[3] J. Duan, H. Wang, H. Li, L. Liu, K. Fan, X. Meng, Z. Zhang, L. Wang, F.-S. Xiao, *EES Catal.* **2023**, *1*, 529–538.

[4] K. Qian, W. Tian, L. Yin, Z. Yang, F. Tian, D. Chen, *Appl. Catal. B: Environ.***2023**, *339*, 123159.

[5] T. M. Ukarde, H. S. Pawar, *Fuel* **2021**, *285*, 119155.

[6] W. Charusiri, N. Phowan, A. Permpoonwiwat, T. Vitidsant, *ACS Omega* **2023**, *8*, 40785–40800.

[7] S. Zhou, P. Li, H. Pan, Y. Zhang, *Ind. Eng. Chem. Res.* **2022**, *61*, 11407–11416.

[8] K. Akubo, M. A. Nahil, P. T. Williams, *J. Energy Inst.* **2019**, *92*, 195–202.

[9] G. Spoto, S. Bordiga, G. Ricchiardi, D. Scarano, A. Zecchina, E. Borello, *J. Chem. Soc., Faraday Trans.* **1994**, *90*, 2827–2835.

[10] F. Geobaldo, G. Spoto, S. Bordiga, C. Lamberti, A. Zecchina, *J. Chem. Soc., Faraday Trans.* **1997**, *93*, 1243–1249.

[11] J. Yi Luo, X. Xian Chen, W. Da Li, W. Yuan Deng, W. Li, H. Yuan Wu, L. Feng Zhu, Q. Guang Zeng, *Appl. Phys. Lett.* **2013**, *102*, 113104.

[12] P. Beato, E. Schachtl, K. Barbera, F. Bonino, S. Bordiga, *Catal. Today* **2013**, *205*, 128–133.

[13] Q. Wang, Y. Li, A. Serrano-Lotina, W. Han, R. Portela, R. Wang, M. A. Bañares, K. L. Yeung, *J. Am. Chem. Soc.* **2021**, *143*, 196–205.

# Author Contributions

Qimin.Zhou: data curation (lead), investigation (lead), writing of original draft (lead)

Weiqiang Gao: data curation (lead), investigation (lead)

Deliang Wang: data curation (lead), formal analysis

Yinlong Chang: data curation (lead), data curation (supporting)

Hanxi Guan: formal analysis (supporting)

Khak Ho Lim: formal analysis (supporting)

Xuan Yang: formal analysis (supporting)

Pingwei Liu: design and supervision of the research, formal analysis, project administration (lead)

Wen-Jun Li: design and supervision of the research

Bo-Geng Li: design and supervision of the research

Qingyue Wang: design and supervision of the research, data curation (lead), project administration (lead), funding acquisition (lead)

All authors discussed the results and commented on the manuscript.
